# Trade credit and firm investments: empirical evidence from Italian cooperative banks

**DOI:** 10.1007/s11156-022-01122-3

**Published:** 2022-12-13

**Authors:** Stefano Filomeni, Michele Modina, Elena Tabacco

**Affiliations:** 1 University of Essex, Essex Business School, Finance Group, Colchester, UK; 2 University of Molise, Campobasso, Italy; 3 Voghera (Pavia), Italy

**Keywords:** SMEs, Trade credit, Investment, Relationship lending, Soft information, Cooperative bank, G21, D22, D82

## Abstract

By exploiting a unique and proprietary panel dataset comprising 6480 Italian SMEs having a relationship with 99 cooperative banks over the period 2008–2014, we investigate the influence of the trade credit channel on firm investment decisions in the Italian market, distinguished by a considerable presence of relationship cooperative banks’ branches with a heterogeneous geographical distribution. Firstly, our findings confirm a significant influence of the trade credit channel on firm investment decisions. Secondly, we document that SMEs located in those Italian provinces with an abundance of cooperative banks’ branches rely less on trade credit to finance investments. Lastly, we show that longer firm-bank relationships decrease firm dependence on trade credit to boost investments. Our study is of particular relevance because it strengthens the effectiveness of the trade credit channel for SMEs in spurring corporate investments. Indeed, fostering a deep understanding of the real effects of firm financing sources is paramount to encourage investment by SMEs and to allow them to preserve their positioning in the market. Moreover, we exploit the Italian market, well-suited to perform such an analysis, since it is characterized by more inter-personal financing relationships as compared to other countries.

## Introduction

In a perfect capital market, trade credit investment and financing decisions are independent because companies enjoy an unlimited access to a wide array of sources of finance in a scenario in which asymmetric information is absent (Modigliani and Miller 1958): companies are always capable to obtain external finance without problems at a reasonable price. However, in a more realistic imperfect capital market, companies may face an opportunity cost associated with trade credit due to the potential difficulty experienced by a firm to access credit through different sources of financing, such as bank credit. This is particularly relevant for small and midsize enterprises (SMEs) for which this opportunity cost is likely to be higher due to their informational opaqueness. Indeed, SMEs have borrowing issues related to information asymmetries and mostly rely upon trade credit and relationship lending based on soft information. This feature not only makes trade credit the only other viable external source to bank credit during their life cycle (Canto-Cuevas et al. 2019), but also raises the likelihood that SMEs will get bank credit-constrained due to greater perceived risk. In this scenario, credit-constrained SME are oriented to turn to trade credit which is the most important alternative to bank lending as a source of external financing (Carbo’-Valverde et al. 2016) in nearly every economy (Demirgüç-Kunt and Maksimovic 2002).^1^ In contrast, larger firms are less likely to fall credit constrained as they tend to borrow from large financial institutions that mostly rely on hard information in their lending decisions (Filomeni et al. 2020, 2021; Stein 2002; Berger and Udell 2006). In this regard, Berger and Udell (2006) show that large institutions tend to lend to larger firms, while small institutions lend more to smaller firms based on stronger bank-firm relationships (Haynes et al. 1999; Cole et al. 2004; Scott 2004; Berger et al. 2005b).

Within this context, this paper provides a novel contribution to the literature by investigating the extent to which SME reliance on the trade credit channel to finance investment decisions is affected by the structure of the local banking system and relationship banking features. To address our research objectives, on the one hand, we focus on SMEs in Italy because they represent the backbone of the Italian economy and typically have fewer options to access capital markets as they are less transparent than their larger corporate counterparts and poses a higher credit risk (Petersen and Rajan 1997; Carey et al. 1993; Berger and Udell 1998). Trade credit plays an important role in SME financing decisions (Ogawa et al. 2013; Martınez-Sola et al. 2014) and it is the only major source of financing (Berger and Udell 1998).^2^ On the other hand, Italy is characterized by geographical heterogeneity in the local banking system across the country: several provinces have an abundance of cooperative banks (Alessandrini and Zazzaro 1999; Alessandrini et al. 2009), mostly relying on soft information in their credit relationships. Indeed, this allows us to exploit within-country geographical variation in the degree of inter-personal financing relationship characterizing the local banking system. At the same time, Italy provides an ideal setting for our study as SMEs represent a large segment of the corporate market (Bonaccorsi di Patti and Gobbi 2001; Guiso et al. 2004; Benfratello et al. 2008; Alessandrini et al. 2009; La Rocca et al. 2010). Therefore, the Italian banking system, characterized by more inter-personal financing relationships compared to other countries, is well-suited to perform our analysis.

In order to address our aforementioned research objectives, firstly, we investigate the real effects of the trade credit channel on long-term investments (Carbò-Valverde et al. 2016; Ferrando and Wolski 2018). We do so by using a panel dataset comprising 6480 Italian SMEs operating with 99 cooperative banks in the period 2008–2014. Following Love et al. (2007), Goncalves et al. (2018), and D’Mello and Toscano (2020), we measure trade credit as the net effect of extended and received trade credit, i.e., net trade credit.^3^ Secondly, we specifically explore whether the local banking system affects the relationship between trade credit and SME investment decisions. We do this by exploiting within-country variation in the different degrees of inter-personal financing relationship of the local banking system, characterized by a heterogeneous geographical distribution of relationship cooperative banks’ branches. Thirdly, the granularity of the data at our disposal allows for the collection of information not only on firm accounting data, but also on specific firm-bank relationship features. In this regard, we study the differential effects of trade credit on firm investments by accounting for firm-bank relationship heterogeneity based on the length of the credit relationship and on the bank-borrower distance. Indeed, longer and closer firm-bank relationships lead to an increased collection of proprietary “soft” information on the part of the bank following repeated interactions with the same firm over time (Petersen and Rajan 1994; Berger and Udell 1995; Uchida et al. 2012; Bolton et al. 2016; Beck et al. 2018). Overall, we believe addressing these questions is of crucial importance for policy makers in order to draw appropriate conclusions on the real effects of the trade credit channel as a financing source alternative to bank lending, and on whether the magnitude of the trade credit effect differs according to specific features of the bank-firm relationship and the local banking system. To the best of our knowledge, this study represents the first attempt to investigate the real effects of the trade channel for SMEs’ investment decisions by exploiting variation in inter-personal financing relationships characterizing the different geographies of the Italian banking market. Moreover, the specificity of the sample, which refers to small businesses financed by local banks, allows us to investigate the phenomenon at a much higher level of detail than it is typically found in the literature.

By way of preview, firstly we find a significant influence of the trade credit channel on firm investment decisions, suggesting that net trade credit significantly affects the growth rate of firm investment. Indeed, an increase in net trade credit triggers a decrease in the year-on-year percentage change of firm investments. This negative effect can be attributed to the liquidity-absorbing consequence of an increase in net trade credit. Secondly, we document that those SMEs located in Italian geographical areas characterized by an abundance of cooperative banks’ branches, relying on soft information-intensive relationship banking, rely less on trade credit to finance their investment decisions. This is supportive of Berger et al. (2005b)’s view that local cooperative banks still benefit from a competitive advantage over nationwide banks in small business lending, since the latter are characterized by organizational complexity and face more severe communication frictions due to the greater distance between their headquarters and local branches. Lastly, we provide evidence that shorter firm-bank relationships lead to a greater dependence of companies on the trade credit channel to boost investments, while firm-bank geographical proximity does not exert a significant influence on this nexus due to the nature of our sample of cooperative banks operating with SMEs mostly on a local basis.

Within the related literature, our paper is closely related to Carbò-Valverde et al. (2016), hereafter referred to as C-V, who analyze for the first time whether trade credit provided an alternative source of external financing for SMEs’ investments during the 2008 crisis by using financial and banking data of nearly 40,000 businesses in Spain over the period 1994–2010. Similarly to C-V’s work, our paper assesses whether bank credit-constrained SMEs turned to trade credit as an alternative source of external financing to boost investments using information on firm characteristics and bank-firm lending relationships. However, our paper differs from C-V’s study by adding new dimensions to the analysis related to relationship banking: it exploits within-country variation in inter-personal financing relationships characterizing the different geographies of the Italian banking market.

Our paper is also close to Ferrando and Wolski (2018) who investigate the relationship between net trade credit and firms’ investment levels by focusing on financially distressed firms. They provide evidence that, while net trade credit has an overall negative impact on investment level due to its liquidity-absorbing effect, this effect is less pronounced for more financially distressed firms since, through capital expenditure, the latter aim at maintaining crucial business relations with their customers in order to participate in the final profits via trade credit repayments. However, even if our results are supportive of the notion that a firm positive net trade credit position is liquidity-absorbing as it reduces the firm growth rate of investments, our paper extends the analysis by contemplating the relevance of the specific features of the bank-firm relationship within the context of trade credit. Moreover, we observe the exact firm-bank relationship, not just relying on a “matching procedure based on a fuzzy-matching algorithm subject to potential biases stemming from spurious firm-bank relations” (Ferrando and Wolski 2018).

Moreover, we add a relationship banking dimension (Giannetti et al. 2011) and focus on bank credit constrained SMEs mostly relying on the trade credit channel for their investment choices (Carbò-Valverde et al. 2016). Unlike the focus of Giannetti et al. (2011) on trade credit and on aspects of bank-firm relationships in the United States, the Italian dimension of our analysis allows us to investigate the impact of trade credit on investment decisions in a more bank-oriented economic context.

This paper contributes to the literature by providing novel evidence on the reliance of Italian SMEs' capital investment on trade credit. Moreover, this study proves that relationship banking provided by Italian cooperative banks weakens the trade credit influence on firm investments. Thus, it adds to the literature new evidence on how bank credit can replace trade credit as a funding source for SMEs. With the crisis generated by Covid-19, our study takes on even greater relevance. The shock that affected the economic world due to Covid-19 was strong. With countries accounting for more than 50% of world GDP frozen for at least two months, the decline in revenues has been more sustained than in previous recessions. The most vulnerable companies are those of smaller size that do not make use of securities markets and for which recourse to government funds is not always easy. Without considering the effects of government support interventions, there is a strong and ongoing concern that SMEs have been hardly hit by the pandemic. In this context, finding adequate solutions to help SMEs is essential given that over two thirds (70%) of global employment is provided by small economic units (ILO 2019). Understanding how trade credit supports corporate investment activity can encourage academics, policy makers and operators to find the most appropriate solutions (e.g., supply chain credit) to support SMEs not only in normal times but also in times of crisis in order to absorb the impact of crisis periods and to continue investing even in uncertain scenarios.

The remainder of this paper is organized as follows. Section 2 presents the Italian banking system with a focus on its geographical heterogeneity, relationship banking, and cooperative banks, Sect. 3 discusses the relevant literature, Sect. 4 describes our data, Sect. 5 presents our empirical methodology, Sect. 6 highlights the results of our empirical analysis, Sect. 7 reports on several robustness tests and, lastly, in Sect. 8 we conclude.

## The Italian banking system: cooperative banks, relationship banking and geographical heterogeneity

Cooperative banks play an important role in the capital markets of many countries (McKillop et al. 2020).^4^ In Italy, today, they are the most representative form of banking localism. Cooperative credit banks have a direct presence in more than a third of the Italian municipalities and, in 620 municipalities (out of 7903), they operate as a single intermediary (Bank of Italy 2019). Their vocation to retail banking is evidenced by the fact that 59% of assets are destined for loans to households and small- and medium-sized businesses. Support for the territory is confirmed by the destination of the collected resources: for every 100 euros of savings collected in the area, 87 euros become credit to the real economy of that area.

The small size and the orientation to the local market favour relationship lending and the reduction of information asymmetries between lenders and borrowers (Petersen and Rajan 1994; Berger and Udell 1995; Elsas 2005; Howorth and Moro 2012; Beck et al. 2018). The information advantage enjoyed by cooperative banks over their larger counterparts, as well as their proximity to the entrepreneurial and social fabric of the territory, translate into a better capacity to select and monitor opaque borrowers such as SMEs (McKillop et al. 2020). The superior ability to collect and manage information relating to customers, especially soft information, has meant that the impact of the financial crisis on the availability of credit disbursed by cooperative banks was less severe than the one observed in different types of banks (Ferri et al. 2014). Indeed, in the last decade the cooperative banks introduced net loans by over €11 billion into the economic circuit with an increase of 9.1% higher than the overall growth in the credit market of + 4.6% (Lopez et al. 2019).

While Italy has been unified for the last 150 years, the local banking system varies notably across provinces. As a matter of fact, several provinces are characterized by an abundance of small cooperative banks that operate in restricted territorial areas (Alessandrini and Zazzaro 1999; Alessandrini et al. 2009). Local cooperative banks benefit from competitive advantages over nationwide banks, as the latter are afflicted by organizational complexity and more severe problems in communicating information (Berger et al. 2005b; Filomeni et al. 2021). Indeed, the distance between the bank headquarters and local branches of nationwide banks gives rise to information frictions within the banking organization, because the bank headquarters are less able to interpret the information coming from distant branches than information from closer ones (Stein 2002; Liberti and Petersen 2019; Filomeni et al. 2020, 2021).

On the one hand, while the lending decisions of large national banks tend to be based on hard information, small cooperative banks rely on soft information collected directly and indirectly through personal bank-firm relationships and continuous interaction with local firms (Howorth and Moro 2006). On the other hand, suppliers provide credit to their customers because the soft information accumulated in repeated trading relationships provides them with a significant advantage over banks in granting credit (Petersen and Rajan 1997). It follows that, if both firm trade credit decisions and local cooperative banks’ credit decisions rely on soft information, trade credit and cooperative bank loans could act as substitutes. Consistent with this view, a high proportion of cooperative bank branches in a province should reduce the need for trade credit and, as such, affect the relationship between trade credit and firm investment decisions. As a matter of fact, a local bank, whose employees are part of the local community, and that may be owned or managed by local community members, possesses a more direct and in-depth knowledge of firms located in its operating area. Indeed, the local bank participates to the local community life, thus collecting information not available to banks that operate at a distance (Angelini et al. 1998; Stein 2002). Moreover, even if nationwide banks’ local branches may integrate borrowers’ hard information with valuable soft information collected locally, or if large complex banks use transaction lending technologies well-suited to SMEs such as credit scoring (Berger and Udell 2006; Ferri and Neuberger 2014), cooperative banks are still expected to benefit from an informational advantage in providing loans to local firms due to their engagement in relationship lending (Bolton et al. 2016; Filomeni et al. 2020, 2021). Bartoli et al. (2013) note that transactional lending, even when using sophisticated technologies, does not substitute for relationship banking in the granting of soft-information intensive loans to SMEs. Presbitero and Zazzaro (2011) prove evidence that the organizational structure of local credit markets influences relationship lending. On the one hand, in markets where large, out-of-market banks predominate, increases in interbank competition are detrimental to relationship lending. On the other hand, in markets with a large group of small local banks, increased competitive pressure from outside pushes banks to further cultivate their relationship ties with customers.

## Literature review and hypotheses development

The relevance of trade credit as an important alternative source to bank finance has not gone unnoticed within the academic community, where several papers have analysed both the financial and economic aspects of trade credit (Pattnaik 2020a) and the multi-disciplinary nature of inter-firm credit transactions (Pattnaik 2020b). In this regard, Pattnaik et al. (2020a) and Pattnaik et al. (2020b) provide a comprehensive and up-to-date overview of the trade credit literature. While the former focuses on the financial and economic aspects of this literature, the latter displays a multidisciplinary nature in line with the evolution and growth of related research. Paul and Boden (2008) recognize that the theoretical and empirical exposure of the trade credit literature is diversified with contributions from multiple streams of studies in order to better investigate the numerous factors influencing the trade credit channel.

From an industrial perspective, product natures (Giannetti et al. 2011), buyer–supplier relationships (McMillan and Woodruff 1999), institutional establishment including the legal system and level of political links (Barrot 2015) influence, among others, trade credit demand and supply. Simultaneously, trade credit channel is guided by the financial infrastructure of an economy (Fisman and Love 2003; Miwa and Ramseyer 2008; Degryse et al. 2018), economic policy uncertainty (D’Mello and Toscano 2020; Jory et al. 2020), financial market development (Ge and Qiu 2007; Abdulla et al. 2017), company financials (Andrieu et al. 2018; García-Teruel and Martínez-Solano 2010b; Dary and James 2019), financing constraints and macro-economic drivers (Bastos and Pindado 2013; Jinjarak 2015; Carbó-Valverde et al. 2016). Beside the quantitative perspectives, qualitative research shows that both cultural (El Ghoul and Zheng 2016; Bedendo et al. 2020) and social factors (Wu et al. 2014; Levine et al. 2018) influence the demand and supply of trade credit. Within an increasingly complex global market, the trade credit channel plays a critical role in driving business growth (Chowdhury and Lang 1996; Fisman and Love 2003) and affects inventory policy (Haley and Higgins 1973; Chung et al. 2005), competitive advantage (Pirttilä et al. 2019), and firm performance (Allen et al. 2019).

For financial theory, trade credit is a financing agreement between non-financial corporations extended to meet the business objectives of firms with or without banking intermediation (Schwartz 1974; Mian and Smith 1992; García-Teruel and Martínez-Solano 2010b). The agreement has a double advantage: the buyer has a financial benefit as it extends the payment deadline; those who sell have a commercial advantage because, thanks to the extension of the terms of collection, they can benefit from an increase in sales. Acknowledging trade credit as a significant source of finance to small and medium enterprises (SMEs), Petersen and Rajan (1997) empirically validate the financing and marketing theories of trade credit and provide some of the methodologies widely followed in later empirical investigations (García-Teruel and Martínez-Solano 2010a; Afrifa and Gyapong 2017). The financing hypothesis dominates in rationalizing the empirical results given that the point at which trade credit replaces bank credit as an alternative source of financing is among those most frequently dealt with (Bastos and Pindado 2013; Carbò-Valverde et al. 2016; Norden et al. 2020).

Among the different dimensions of trade credit research, we aim at addressing the following research questions summarized in the hypotheses described in the next sub-sections, with the objective to broaden the vision of the work on the financial perspective of the trade credit channel:

- Is there a significant relationship between trade credit and SME investment decisions?
- If so, does this relationship vary in provinces with an abundance of relationship cooperative banks’ branches?
- If so, is this relationship affected by firm-bank relationship features?

### Trade credit channel and firm investment decisions

The empirical evidence on the influence of the trade credit channel on firm investment decisions is still mixed. While Coricelli and Frigerio (2019) find that the provision of trade credit may drain the investment-supportive liquidity, Dass et al. (2015) provide theoretical and empirical evidence that the provision of trade credit can act as a commitment device for making relationship-specific investments.^5^ Consistent with Coricelli and Frigerio (2019), Ferrando and Wolski (2018) study the relationship between net trade credit and firms’ investment levels by focusing on both financially distressed firms and crisis periods by using a large panel of more than 10 million firms in 23 EU countries over the period 2004–2014. Their results suggest that net trade credit has an overall negative impact on firm investment due to its liquidity-absorbing nature. Moreover, Carbò-Valverde et al. (2016) examine whether trade credit provides an alternative source of external finance to SMEs during the crisis. Using firm-level Spanish data they document that credit-constrained SMEs depend on trade credit, but not bank loans, and that the intensity of this dependence increased during the 2008 financial crisis.

Our above discussion on the effects of the trade credit channel on firm investment can be summarized in our first hypothesis (H1):

#### H1

*The trade credit channel significantly affects corporate investment and, as such, brings over real effects in the economy.*

### Trade credit channel and the local banking system

As described in Sect. 2, the structure of the Italian local banking system is characterized by geographical heterogeneity. Its effect on the relationship between trade credit and firm investment is not clear a priori. On the one hand, a greater proportion of local relationship cooperative banks’ branches in Italian provinces should facilitate access to bank loans and reduces finance constraints for SMEs with which they have established relationships in the operating area (Alessandrini et al. 2009; La Rocca et al. 2010). This leads us to expect that facilitated access to bank credit should weaken firm reliance on the trade credit channel to finance investments, if the given firm is located in a province with a high proportion of relationship cooperative banks’ branches. Indeed, local cooperative banks benefit from a competitive advantage over nationwide banks in small business lending, since the latter are characterized by organizational complexity and face more severe communication frictions due to the greater distance between their headquarters and local branches (Berger et al. 2005b). On the other hand, a number of studies challenge this conventional paradigm according to which cooperative banks (small, single-market, local institutions) form strong relationships with informationally opaque small businesses (Haynes et al. 1999; Cole et al. 2004; Scott 2004; Berger et al. 2005b). These studies document that changes in lending technologies and deregulation of the banking industry have made it easier for large and nonlocal banks to serve small, opaque firms (Berger et al. 2014). Berger and Udell (2006) highlight that large banks are also capable of serving small and opaque firms well using hard-information technologies, i.e., credit scoring and lending against fixed asset collateral, consistent with Frame et al. (2001) and Berger et al. (2005a). de la Torre et al. (2010) find that both large and small banks cater to small firms with an increasing use of hard information-based technologies (Berger and Black 2011; Berger et al. 2011). Both Frame et al. (2004) and DeYoung et al. (2011) document that small business credit scoring is accountable for an increase in lending distance.

Our second hypothesis (H2) summarizes the influence of the structure of the local banking system on the nexus between trade credit and firm investment decisions:

#### H2

*The effect of the trade credit channel on firm investments is affected by the proportion of local cooperative banks’ branches relying on soft information-intensive relationship banking.*

### Trade credit channel and relationship banking

Studies investigating the nexus between trade credit and relationship banking are limited. In this regard, a valuable contribution is provided by Giannetti et al. (2011) who document that “trade credit usage is correlated with the buyer’s banking relationships”*,* by focusing on the United States*.* Specifically, they show that firms receiving trade credit secure financing from relatively uninformed banks, thus supporting our findings that the proprietary soft information collected by the bank decreases the likelihood of the borrowing firm being credit-constrained (Berger and Udell 2002; Gobbi and Sette 2014; Presbitero et al. 2014; Bolton et al. 2016; Casu et al. 2022). Moreover, Giannetti et al. (2011) find that firms that make greater use of the trade credit channel have shorter relationships with their banks. In a similar vein, Von Thadden (1995), from a theoretical point of view, and Petersen and Rajan (2002), under an empirical profile, indicate that firms borrowing from distant banks for short periods are generally considered to have arm’s length relations with their lenders, while Degryse and Ongena (2007) provide evidence that the choice between relationship versus transactional banking does not depend on the level of local banking competition. Furthermore, McMillan and Woodruff (1999), Johnson et al. (2002), and Uchida et al. (2013) document that longer duration of trading relationships is often associated with a greater use of the trade credit channel.

Our third hypothesis (H3) summarizes the influence of the trade credit channel on firm investment decisions according to relationship banking-related features:

#### H3

*The effect of the trade credit channel on firm investments is decreasing in the length of the firm-bank relationship, while it is increasing in the bank-firm distance as cooperative banks operate with SMEs mostly on a local basis.*

## Data

This paper exploits the granularity and the uniqueness of a proprietary dataset combining public firm and bank-level financial information with private bank-firm lending information on a sample of 6480 Italian SMEs having a relationship with 99 cooperative banks over the period 2008–2014.

Public financial information concerns the economic and financial characteristics of the companies and banks in our sample. The firm-level variables concern the composition of assets and liabilities, the intensity of the corporate investment activity, the economic performance measured by the return on assets (ROA). The bank-level characteristics concern the profitability (i.e., return on average asset—ROAA), capitalization (i.e., bank's equity ratio), and the quality of the loans disbursed (impaired loans over total loans) by the cooperative banks in our sample. Cooperative banks, which cover about 7.2% of the Italian loan market (McKillop et. al. 2020), are known to be close to the territory and attentive to the needs of smaller companies (Baccarani et al. 2013), such as the SMEs populating our dataset. We collect data on firm accounting variables from Centrale dei Bilanci (CEBI) and on bank variables from Bankscope.^6^

Private lending information is of two types. On the one hand, we observe the duration of the credit relationship and the geographical distance between the borrowing firm and the lending bank. The data were provided exclusively to us by CSD, an Italian company that manages the information system of more than 100 Italian cooperative banks. On the other hand, we collect data on a borrower’s debt position towards the banking system from the Italian Credit Register (CR) managed by the Bank of Italy.

Our setting involves the presence of 99 cooperative banks located in 100 Italian provinces spread over the Italian territory and lending to 83 different industries according to the 2-digit Ateco industry classification.^7^ In particular, firms in our sample belong to the following six macro-industries: agriculture, commerce, transports and hotels, manufacturing, building and services. We exclude public administration and financial firms. Companies are segmented on the basis of their synthetic code of economic activity, i.e., 2-digit Ateco industry classification, that allows each borrowing company to be correctly associated with a specific sector. Moreover, regarding firm legal form, the fact that all the companies in our sample belong to the capital companies segment makes it possible to have a homogeneous set of accounting data drawn from their annual financial statements.

Lastly, data on the number of cooperative banks’ branches located in Italian provinces are collected from the Bank of Italy.

Table 1 reports the definition of the dependent and explanatory variables used in the analysis and their descriptive statistics.

**Table 1:** Variables’ descriptions and descriptive statistics

Variable	Description	Obs	Mean	Std. dev	Min	Max
*Dependent variable*						
ΔCAPEX	Year-on-year change in capital expenditure	52,913	0.101473	0.427132	− 0.4	1.5
*Firm-level variables*						
NTC	Net trade credit measured as (Accounts Receivable – Accounts Payable)/Total Assets	79,655	0.123351	0.238352	− 0.3355856	0.5961828
CR	Firm current ratio	80,715	120.4969	97.69209	6.4	400
ULC	Firm unit labor cost measured as salary per hour expressed in Euros	71,071	21.17461	16.24462	2.04	62.33
Bank debt/total liabilities	Short- and long-term bank debt over total liabilities (% points)	76,262	40.05312	24.64493	3.88	92.24
Inventory period	Firm's inventory period	63,335	164.405	299.3785	1.78	1243.05
Financial fixed assets/total assets	Ratio of the firm's financial fixed assets to total assets (% points)	50,881	0.069644	0.126612	0.0003037	0.4853714
Intangible assets/total assets	Ratio of the firm's intangible assets to total assets (% points)	61,714	4.175309	6.366742	0.04	23.95
Tangible assets/total assets	Ratio of the bank's firm tangible assets to total assets (% points)	76,856	25.34375	23.92339	0.71	80.71
Inventory/total assets	Ratio of the firm's inventory to total assets (% points)	66,260	26.77742	26.16904	0.49	90.23
Total assets	Firm total assets	87,527	5157.792	8127.442	68,000	31,449,000
ROA	Firm's return on assets	84,027	1.865897	7.70142	− 14.14	22.41
DPO	Firm's days payable outstanding	79,999	177.6105	179.2663	24.54629	771.1868
DSO	Firm's days sales outstanding	80,516	179.8311	156.6654	17.69697	673.3621
*Relationship banking variables*						
Length of relationship	Number of years of the bank-firm relationship [logarithm of 1 + length of relationship (years)]	54,017 [54,017]	6.864172 [1.66431]	6.967365 [0.95776]	0 [0]	54 [4.0073]
Borrower-to-branch distance	Binary variable = 1 if the borrower and the bank are located in the same geographical location, and 0 otherwise	52,569	0.648329	0.477497	0	1
*Bank-level variables*						
Bank ROAA	Bank's return on average assets	82,196	0.211137	0.658931	− 2.854	1.475
Bank equity ratio	Bank's equity ratio	82,196	9.262379	2.438896	2.416	19.017
Bank impaired/total loans	Ratio of the bank's impaired loans over gross loans	79,676	9.089273	5.855901	1.434	40.668
Bank total assets	Log of the bank's total assets	82,196	14.00308	0.723207	11.05406	16.02128
Cooperative banking	Binary variable equal to 1 if the density of cooperative banks’ branches measured by the number of cooperative branches located in a province over the number of firms operating in that province is above the median value of the distribution, and 0 otherwise [Density of cooperative banks’ branches]	54,045 [54,045]	0.49 [0.002]	0.50 [0.001]	0 [0]	1 [0.009]
Commercial banking	Binary variable equal to 1 if the density of commercial banks’ branches measured by the number of commercial branches located in a province over the number of firms operating in that province is above the median value of the distribution, and 0 otherwise [Density of commercial banks’ branches]	54,045 [54,045]	0.48 [0.009]	0.50 [0.002]	0 [0.004]	1 [0.016]

Data collected from CSD, the Italian Credit Register (CR), and the Bank of Italy

## Empirical methodology

The empirical approach relies on panel data estimation on a sample of 6480 Italian SMEs having a relationship with cooperative banks in the period 2008–2014. Specifically, each SME in our sample is observed over multiple time periods and has a credit relationship only to the cooperative bank considered as its main financier. Our panel data structure allows us to control for time invariant and unobserved factors specific to each firm-bank pair driving differences in firm investment decisions. That is, bank-firm (pair) fixed effects are included in all regressions. The estimated models are saturated by time and industry-specific fixed effects, or by a vector of industry-year fixed effects with industries characterized at the 2-digit level of the Ateco 2007 classification of economic activities.^8^

In model selection, we performed the Hausman test to determine whether to implement a fixed- or random-effects model. This test leads us to reject the null hypothesis of random effects, thereby accepting the implementation of a fixed-effect (FE) model. To mitigate the issue of the heteroskedasticity of residuals, detected by performing the modified Wald test for groupwise heteroskedasticity in FE regression models, we test all our models using heteroskedasticity-robust standard errors clustered at the bank-firm (pair) level. To avoid the effect of outliers driving our results, data are winsorized at the 5% level.^9^

### Trade credit channel and firm investment decisions

Firstly, we test whether the trade credit channel affects firm investment. Following Kaplan and Zingales (1997), Fazzari et al. (2000), and Carbo’-Valverde et al. (2016), firm investment is introduced as the ratio of the year-on-year change in capital expenditure relative to the total amount of capital of the previous year, where capital expenditure is computed as the annual change in fixed assets, i.e., inclusive of financial, intangible and tangible fixed assets, plus amortization and depreciation ($\Delta CAPEX$). Since in our sample each SME only connects to the main financing cooperative bank, the dependent variable $\Delta CAPEX$ is denoted at the bank-firm pair level.

Following Afrifa and Gyapong (2017), the trade credit channel is introduced as the difference between the firm’s account receivables and account payables scaled by the firm’s size as measured by total assets, i.e., net trade credit, *NTC*. Specifically, the net trade credit position measures the cash tied up in the trade cycle before it comes back out as cash again. The longer the net trade credit position of a given firm, the greater is its working capital requirement (Bernstein and Wild 1998). On the one hand, a positive net trade credit position requires the company to finance the days taken by account receivables to be cashed in by tapping its financing sources. On the other hand, a negative net trade credit position reflects a situation in which the given firm is being paid for its sales before having to pay its account payables. Following Petersen and Rajan (1997), when we view the firm as a supplier, “its accounts receivable are a proxy for how much it lends its customers”, while when we view the firm as a customer, “its accounts payable are its borrowing from its supplier”. In this perspective, we construct a measure of the firm net trade credit position by examining commercial relationships cultivated by a firm in the role of both customer (borrower) and supplier (lender) according to the above definitions. Net trade credit is taken at time *t* rather than *t* − 1 to mimic the short-term nature of trade credit contracts, usually less than one year (Ferrando and Wolski 2018).

Our fixed effects (FE) panel data baseline regression model for the trade credit channel and firm investment decisions takes the following form:

1 $$\begin{aligned} \Delta CAPEX_{i,k,n,t} = & \;\alpha + \beta_{1} NTC_{i,k,n,t} + \mathop \sum \limits_{j = 1}^{z} \delta_{j} X_{i,k,n,t} + \varphi_{i, k} + \gamma D_{industry\;n} \\ & + \;\delta D_{year\;t} + \mu D_{industry\;n} *D_{year\;t} + \varepsilon_{i,k,n,t} \\ \end{aligned}$$

where $X_{i,k,n,t}$ is the vector of control variables representing firm-specific characteristics for firm *i*, having a credit relationship with bank *k*, and operating in industry *n* in year *t* described in Sect. 5.4; $D_{industry\;n}$ are industry dummies to control for industry-specific effects according to the 2-digit Ateco industry classification; $D_{year\;t}$ are yearly time dummies to control for time-specific effects; and $\varepsilon_{i,k,n,t}$ is the error term for firm *i* in year *t;* $D_{industry\;n} * D_{year\;t}$ are industry-year fixed effects in order to control for all time-varying shocks at the industry level. Moreover, the nature of the panel dataset with fixed effects ($\varphi_{i, k}$) allows us to control for firm *i*-bank *k* pair-specific unobserved heterogeneity.

A description of the construction of the model variables, as well as their descriptive statistics, is presented in Table 1.

### Trade credit channel and the local banking system

Secondly, we explore whether the structure of the local banking system affects the relationship between trade credit and SME investment decisions. That is, we explore whether a high proportion of cooperative banks’ branches in a province should reduce the need for trade credit and, as such, moderate the relationship between trade credit and firm investment decisions. To this purpose, we interact our measure of net trade credit, i.e., *NTC*, with the variable *cooperative banking,* a binary variable equal to 1 if the density of cooperative banks’ branches with respect to the number of firms operating in the Italian province where the SME is headquartered is above the median value of the distribution and 0 otherwise.^10^

Our FE panel data regression model for the trade credit channel and the local banking system takes the following form:

2 $$\begin{aligned} \Delta CAPEX_{i,k,n,t} = & \;\alpha + \beta_{1} NTC_{i,k,n,t} + \beta_{2} NTC_{i,k,n,t} *cooperative\;banking_{p} \\ & + \;\beta_{3} \;cooperative\;banking_{p} + \;\mathop \sum \limits_{j = 1}^{z} \delta_{j} X_{i,k,n,t} + \varphi_{i, k} + \gamma D_{industry\;n} \\ & + \;\delta D_{year\;t} + \mu D_{industry\;n} * D_{year\;t} + \varepsilon_{i,k,n,t} \\ \end{aligned}$$

where $X_{i,k,n,t}$ is the vector of control variables representing firm-specific characteristics for firm *i*, having a credit relationship with bank *k*, and operating in industry *n* in year *t* described in Sect. 5.4; $cooperative\;banking_{p}$ reflects the density of cooperative banks’ branches with respect to the number of firms operating in the given Italian province *p*.

By investigating the sign and the statistical significance of the coefficient $\beta_{2}$ in Eq. (2) we can make inferences on whether the structure of the local banking system affects firm reliance on the trade credit channel to spur investments. On the one hand, a positive and statistically significant coefficient $\beta_{2}$ would document that a high proportion of relationship cooperative banks’ branches should facilitate small business access to bank credit and, as such, weaken firm reliance on the trade credit channel to spur investments. On the other hand, a non-statistically significant coefficient $\beta_{2}$ would signal that changes in lending technologies and deregulation of the banking industry have made it easier for large and nonlocal banks to serve small, opaque firms, thus eroding the comparative advantage of local soft information-intensive relationship banks in serving small businesses.

### Trade credit channel and relationship banking

Thirdly, we investigate whether the influence of the trade credit channel on firm investment decisions is moderated by firm-bank relationship features.^11^ Specifically, we are interested in investigating whether the specific characteristics of a given firm-bank financing relationship lead to a differential impact of the trade credit channel on firm investments. To this purpose, we construct the variable *relationship banking* that is proxied by either the length of the bank-firm relationship (*length of relationship)* or the geographical distance between the borrowing firm and the lending branch (*borrower-to-branch distance)*. *Relationship banking* varies at the bank-firm-time level for *length of relationship* and at the bank-firm level for *borrower-to-branch distance*.

Our FE panel data regression model for the trade credit channel and relationship banking takes the following form:

3 $$\begin{aligned} \Delta CAPEX_{i,k,n,t} = & \;\alpha + \beta_{1} NTC_{i,k,n,t} + \beta_{2} NTC_{i,k,n,t} *relationship\;banking_{i,k,n,t} \\ & + \;\beta_{3} \;relationship\;banking_{i,k,n,t} + \;\mathop \sum \limits_{j = 1}^{z} \delta_{j} X_{i,k,n,t} + \varphi_{i, k} + \gamma D_{industry\;n} \\ & + \;\delta D_{year\;t} + \mu D_{industry\;n} * D_{year\;t} + \chi D_{bank\;k} + \varepsilon_{i,k,n,t} \\ \end{aligned}$$

where $X_{i,k,n,t}$ is the vector of control variables representing firm-specific characteristics for firm *i*, having a credit relationship with bank *k*, and operating in industry *n* in year *t* described in Sect. 5.4, and *χD*_*bank k*_ represents bank fixed effects.

Therefore, at the firm-bank relationship level, we observe the length of the bank-firm relationship and the bank-borrower distance (Agarwal 2010; Agarwal and Hauswald 2010; Alessandrini et al. 2009; Carbo’-Valverde et al. 2016; Filomeni et al. 2020, 2021).^12^ The motivation lays in the fact that, a priori, firm characterized by deeper banking relationships might decrease their reliance on the trade credit channel as a funding source in a context where relationship banking provides benefits for both the lender and the borrower in terms of a Pareto-improving exchange of information between the parties involved and several welfare-improving contractual features.

Firstly, the length of the bank-firm relationship allows us to assess the nature and the strength of the bank-firm relationship, which facilitates the collection of borrower-specific information, and it is defined as a categorical variable *length of relationship* equal to number of years of the lending relationship. To test whether the influence of the trade credit channel on firm investment is negatively associated to the length of the bank-firm relationship, we interact *NTC* with *length of relationship* and investigate the sign and the significance of coefficient $\beta_{2}$ in Eq. (3) associated with the interaction term. As anticipated, *length of relationship* allows us to capture the nature and the strength of the bank-firm relationship which might significantly affect the relationship between trade credit and firm investment behavior.

Secondly, the bank-borrower distance is assessed by defining the binary variable *borrower-to-branch distance* equal to one if the borrower’s headquarters and the branch in which the loan officer in charge of developing the firm-bank relationship are located in the same geographical location, and 0 otherwise. Since relationship lending is considered an appropriate tool for bank lending to more informationally opaque SMEs and evidence suggests that it could alleviate credit constraints (Berger and Udell 2002; Scott 2006; Gobbi and Sette 2014; Presbitero et al. 2014; Bolton et al. 2016; Bose et al. 2021), it potentially mitigates firm reliance on the trade credit channel as a financing source. To test whether the influence of the trade credit channel on firm investment significantly differs according to the bank-borrower distance, we interact *NTC* with *borrower-to-branch distance* and investigate the sign and the significance of coefficient $\beta_{2}$ in Eq. (3) associated with the interaction term. As anticipated, *borrower-to-branch distance* allows us to assess the ease of soft information transmission between the borrowing firm and the lending bank due to greater geographical proximity.

### Control variables

In our regressions, we control for several firm-specific characteristics that could influence firm investment behaviour, other than the trade credit channel.

At the firm level, we control for the firm current ratio $\left( {CR_{i,n,t} } \right)$*,* measured as the ratio of current assets over current liabilities, in order to supervise the effect that the hedging of current assets may have on firm investment changes. We also control for the firm unit labor cost $\left( {ULC_{i,n,t} } \right)$ as labor costs may divert cash flows away from investment purposes, for the firm inventory period $(Inventory_{i,n,t} )$ as the longer the inventory is held, the more the warehouse drains financial resources and reduces the firm investment possibilities, for the firm inventory to assets ratio $\left( {Inventory/Total\;Assets_{i,n,t} } \right)$ which reflects the portion of assets tied up in inventory since the inventory changes are associated with the release of cash flows which promotes investments, and for the firm return on assets $\left( {ROA_{i,n,t} } \right)$ to take into account the influence of firm profitability on investment decisions. Finally, we include three different additional ratios, i.e., the firm tangible assets to total assets ratio $\left( {Tangible\;Assets/Total\;Assets_{i,n,t} } \right)$, the firm intangible assets to total assets ratio $\left( {Intangible\;Assets/Total\;Assets_{i,n,t} } \right)$, the firm financial fixed assets to total assets ratio $\left( {Financial\;Fixed\;Assets/Total\;Assets_{i,n,t} } \right)$.

At the firm-bank level, we control for the short term and long term bank debt scaled by total liabilities $\left( {Bank\;Debt/Total\;Liabilities_{i,k,t} } \right)$, in line with Coluzzi et al. (2012) and Heshmati (2001) who show that access to bank loans is an important driver of firm growth and may, consequently, affect firm investment behavior.

## Results

**Fig. 1:**
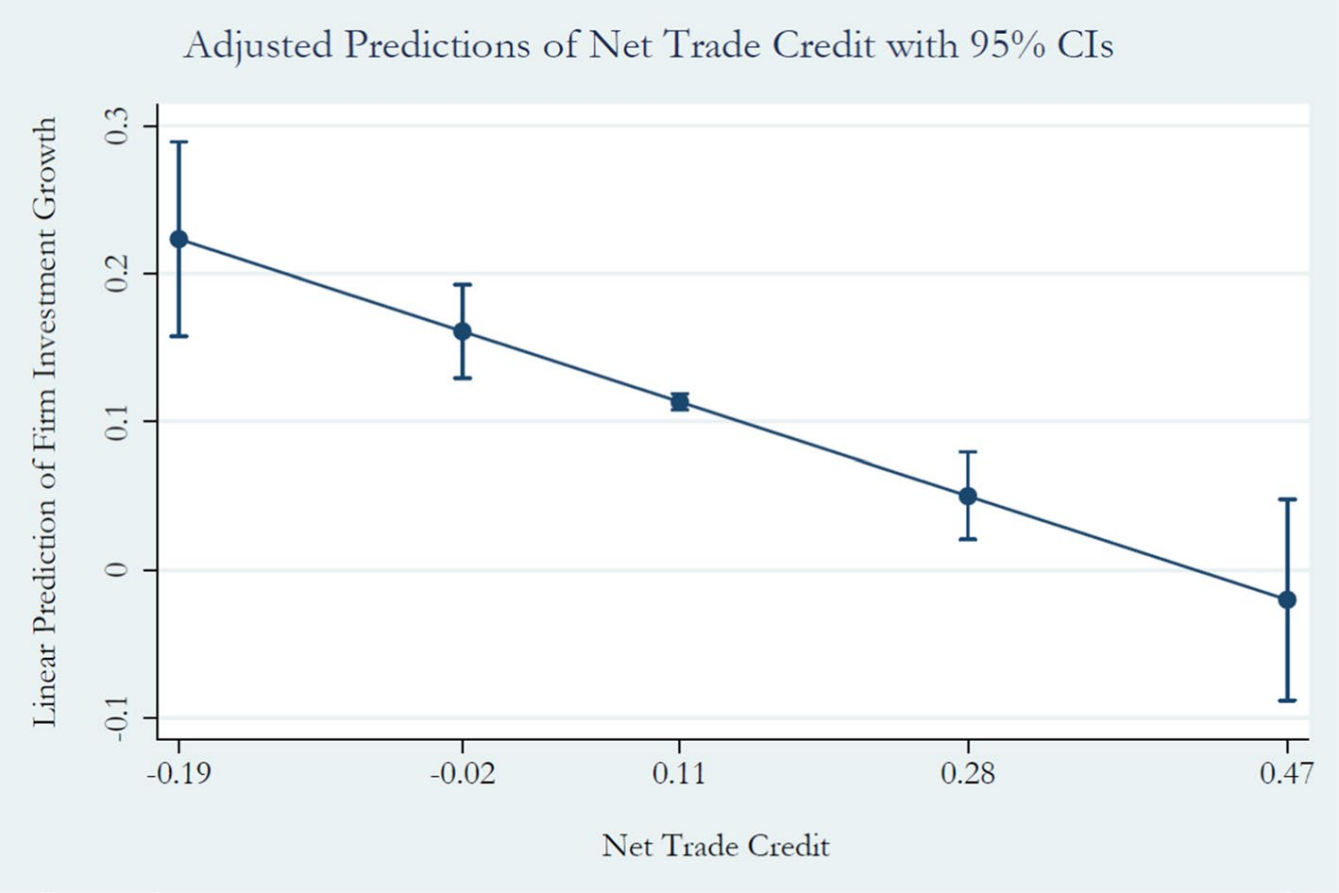
Predicted outcomes of *∆CAPEX* at percentiles of *NTC*. On the x-axis are reported the 10th, 25th, 50th, 75th, and 95th percentiles of *NTC* distribution

Firstly, we are interested in investigating the relationship between the trade credit channel and firm investment decisions. The former is measured by the firm net trade credit position in given year *t* (*NTC*). The latter, our dependent variable, is defined as the ratio of the year-on-year change in capital expenditure between the previous year *t* − 1 and the current year *t* relative to the total amount of capital in the previous year *t* − 1 (*∆CAPEX*). Table 2 shows the results from running our baseline model, which is empirically defined in Eq. (1). We are interested in investigating the statistical significance of the coefficient $\beta_{1}$ associated with our main regressor (*NTC*) to analyse whether the trade credit channel significantly influences firm investment behavior. In this regard, columns (1)–(6) in Table 2 show that the *NTC* coefficient $\beta_{1}$ is negative and statistically significant at the 1% level in all model specifications. We interpret this result to mean that an increase in the firm net trade credit position is reflected in a decrease in the growth rate of capital expenditure, ceteris paribus. Confirmatory graphical evidence is provided by Fig. 1 showing the predicted outcomes of *∆CAPEX* at different percentiles of *NTC:* when moving from the 10th percentile (− 19%), characterizing a firm receiving trade credit, to the 90th percentile of *NTC* distribution, reflecting a firm extending trade credit (46.8%), *∆CAPEX* decreases, on average, from 22.3 to − 2%, respectively.^13^

**Table 2:** Baseline model of trade credit

Variables	(1)	(2)	(3)	(4)	(5)	(6)
	FE panel model	FE panel model	FE panel model	FE panel model	FE panel model	FE panel model
NTC	− 0.526***	− 0.547***	− 0.518***	− 0.299***	− 0.397***	− 0.391***
	(0.054)	(0.054)	(0.054)	(0.053)	(0.066)	(0.066)
ULC					− 0.005***	− 0.005***
					(0.001)	(0.001)
Bank debt/total liabilities					− 0.000	− 0.000
					(0.001)	(0.001)
Financial fixed/total assets				1.278***	1.219***	1.214***
				(0.150)	(0.168)	(0.157)
Intangible assets/total assets				0.021***	0.020***	0.022***
				(0.002)	(0.002)	(0.002)
Tangible assets/total assets				0.014***	0.013***	0.014***
				(0.001)	(0.001)	(0.001)
Inventory/total assets					− 0.003***	− 0.004***
					(0.001)	(0.001)
ROA					0.005***	0.005***
					(0.001)	(0.001)
Observations	16,328	16,328	16,328	16,328	16,328	16,328
R-squared	0.05	0.02	0.09	0.15	0.10	0.15
Number of firm-bank pairs	6550	6550	6550	6550	6550	6550
Yearly FE	Yes	No	No	No	Yes	No
Industry FE	No	Yes	No	No	Yes	No
Year × industry FE	No	No	Yes	Yes	No	Yes

The table presents the results of the FE panel regression analysis for the Trade Credit & Firm Investment Decisions model where the dependent variable is $\Delta CAPEX_{i,k,n,t}$ which is the annual percentage change in the firm’s capital expenditure. $NTC_{i,k,n,t}$ represents the trade credit channel measured as the difference between the firm’s account receivables and account payables (i.e., net trade credit) scaled by the firm’s size as measured by total assets. All variables are defined in Table 1. Columns (1)–(4) are reduced forms of the full model of Eq. (1). Year, Industry and Year*Industry fixed effects are incorporated in regressions where indicated (not reported). Robust errors, reported in parentheses, are clustered at the bank-firm (pair) level

***p < 0.01, **p < 0.05, *p < 0.1, respectively

Secondly, we explore whether the structure of the local banking system affects the relationship between trade credit and SME investment decisions. We do this by exploiting within-country variation in the Italian local banking structure characterized by different degrees of inter-personal financing relationships. As described in Sect. 2, the Italian banking system is populated by a large number of cooperative banks that operate in restricted territorial areas, mostly located in the north part of the country (Alessandrini and Zazzaro 1999; Alessandrini et al. 2009) and that benefit from competitive advantages over nationwide banks by more reliably processing soft information collected directly and indirectly through personal bank-firm relationships (Howorth and Moro 2006). Indeed, nationwide banks are more afflicted by organizational complexity and communications frictions in hardening and transmitting soft information over greater distance between their headquarters and local branches (Berger et al. 2005a, b; Filomeni et al. 2020). This prompts them to research economies of scale in the processing of hard information and to specialize in transactional lending (Ferri and Neuberger 2014). Model specification is shown by Eq. (2). The estimation results are reported in columns (1)–(2) in Table 3. The sign and the statistical significance of the coefficient $\beta_{2}$ on the interaction terms NTC*cooperative banking in both column (1) and (2) is positive and statistically significant, thus suggesting that a high proportion of relationship cooperative banks’ branches facilitates small business access to bank credit and, as such, weakens firm reliance on the trade credit channel to spur investments. This is supportive of the notion that local banks still benefit from a competitive advantage over nationwide banks in small business lending (Berger et al. 2005b), despite the changes in lending technologies and deregulation of the banking industry that are challenging their advantage to serve small, opaque firms (Berger et al. 2014).

**Table 3:** Trade credit & cooperative banking

Variables	(1)	(2)
	FE panel model	FE panel model
NTC	− 0.503***	− 0.499***
	(0.081)	(0.080)
NTC * cooperative banking	0.190***	0.193***
	(0.082)	(0.080)
Cooperative banking	0.001	− 0.001
	(0.025)	(0.025)
ULC	− 0.005***	− 0.005***
	(0.001)	(0.001)
Bank debt/total liabilities	− 0.000	− 0.000
	(0.001)	(0.001)
Financial fixed/total assets	1.224***	1.218***
	(0.168)	(0.156)
Intangible assets/total assets	0.020***	0.022***
	(0.002)	(0.002)
Tangible assets/total assets	0.013***	0.014***
	(0.001)	(0.001)
Inventory/total assets	− 0.003***	− 0.004***
	(0.001)	(0.001)
ROA	0.005***	0.005***
	(0.001)	(0.001)
Observations	16,224	16,224
R-squared	0.11	0.16
Number of firm-bank pairs	6509	6509
Yearly FE	Yes	No
Industry FE	Yes	No
Year × industry FE	No	Yes

The table presents the results of the FE panel regression analysis for the Trade Credit & Local Banking System model where the dependent variable is $\Delta CAPEX_{i,k,n,t}$ which is the annual percentage change in the firm’s capital expenditure. $NTC_{i,k,n,t}$ represents the trade credit channel measured as the difference between the firm’s account receivables and account payables (i.e., net trade credit) scaled by the firm’s size as measured by total assets. All variables are defined in Table 1. Year, Industry and Year*Industry fixed effects are incorporated in regressions where indicated (not reported). Robust errors, reported in parentheses, are clustered at the bank-firm (pair) level

***p < 0.01, **p < 0.05, *p < 0.1, respectively

Thirdly, we investigate whether the influence of the trade credit channel on firm investment decisions differs according to relationship banking features, as described below. Model specification is shown by Eq. (3). Table 4 shows the results of this analysis.

Firstly, to test whether the influence of the trade credit channel on firm investment significantly differs according to the length of the bank-firm relationship, we interact *NTC* with *length of relationship* which is equal to number of years of the lending relationship. Model specification is shown by Eq. (3). The estimation results of our model are reported in columns (1)-(2) in Table 4. The sign and the statistical significance of the coefficient $\beta_{2}$ on the interaction term NTC*length of relationship is positive and statistically significant, as shown in columns (1) and (2) in Table 4, thus suggesting that longer bank-firm relationships are associated with a decreased influence of the trade credit channel on firm investment decisions. These results are supportive of the extant literature providing evidence that proprietary “soft” information, i.e., relationship banking, decreases the likelihood of the borrowing firm being credit-constrained (Berger and Udell 2002; Scott 2006; Gobbi and Sette 2014; Presbitero et al. 2014; Bolton et al. 2016).

Secondly, to test whether the influence of the trade credit channel on firm investment significantly differs according to the bank-borrower distance, we interact *NTC* with *borrower-to-branch distance* which is equal to one if the borrower’s headquarters and the branch in which the loan officer in charge of developing the firm-bank relationship are located in the same geographical location, and 0 otherwise. Model specification is shown by Eq. (3). The estimation results of our model are reported in columns (3)-(4) in Table 4. Consistent with the evidence provided by Alessandrini et al. (2009) on Italian firms, the sign and the statistical significance of the coefficient $\beta_{2}$ on the interaction term NTC*borrower-to-branch distance is not statistically significant meaning that smaller borrower-to-branch distance does not always enhance credit availability. This result is motivated by the nature of our sample of cooperative banks operating with SMEs mostly on a local basis. Nevertheless, the main effect of *NTC* remains in line with our previous results as its coefficient is still negative and statistically significant at the 1% level.

**Table 4:** Trade credit & relationship banking

Variables	(1)	(2)	(3)	(4)
	FE panel model	FE panel model	FE panel model	FE panel model
NTC	− 0.488***	− 0.466***	− 0.483***	− 0.474***
	(0.087)	(0.087)	(0.097)	(0.095)
NTC * length of relationship	0.012**	0.010*		
	(0.006)	(0.006)		
Length of relationship	− 0.120***	0.009*		
	(0.025)	(0.005)		
NTC * borrower-to-branch distance			0.118	0.117
			(0.110)	(0.109)
Borrower-to-branch distance			− 0.255	− 0.325
			(0.211)	(0.312)
ULC	− 0.005***	− 0.005***	− 0.005***	− 0.005***
	(0.001)	(0.001)	(0.001)	(0.001)
Bank debt/total liabilities	− 0.000	− 0.000	− 0.000	− 0.000
	(0.001)	(0.001)	(0.001)	(0.001)
Financial fixed/total assets	1.220***	1.204***	1.215***	1.210***
	(0.168)	(0.156)	(0.172)	(0.161)
Intangible assets/total assets	0.020***	0.022***	0.020***	0.022***
	(0.002)	(0.002)	(0.002)	(0.002)
Tangible assets/total assets	0.013***	0.014***	0.013***	0.013***
	(0.001)	(0.001)	(0.001)	(0.001)
Inventory/total assets	− 0.003***	− 0.004***	− 0.003***	− 0.004***
	(0.001)	(0.001)	(0.001)	(0.001)
ROA	0.005***	0.004***	0.005***	0.005***
	(0.001)	(0.001)	(0.001)	(0.001)
Observations	16,166	16,166	15,576	15,576
R-squared	0.10	0.16	0.11	0.16
Number of firm-bank pairs	6499	6499	6220	6220
Bank FE	Yes	Yes	Yes	Yes
Yearly FE	Yes	No	Yes	No
Industry FE	Yes	No	Yes	No
Year × industry FE	No	Yes	No	Yes

The table presents the results of the FE panel regression analysis for the Trade Credit & Relationship Banking model where the dependent variable is $\Delta CAPEX_{i,k,n,t}$ which is the annual percentage change in the firm’s capital expenditure. $NTC_{i,k,n,t}$ represents the trade credit channel measured as the difference between the firm’s account receivables and account payables (i.e., net trade credit) scaled by the firm’s size as measured by total assets. All variables are defined in Table 1. Bank, Year, Industry and Year*Industry fixed effects are incorporated in regressions where indicated (not reported). Robust errors, reported in parentheses, are clustered at the bank-firm (pair) level

***p < 0.01, **p < 0.05, *p < 0.1, respectively

## Robustness tests

To confirm our empirical results on the relationship between the trade credit channel and firm investment behavior, we perform several robustness checks that leave our previous findings unaffected.

### Influence of crisis periods

As an additional robustness check, firstly we now run our regression models in Eqs. (1), (2), and (3) on the entire sample period by removing from the analysis those years characterized by acute financial instability, i.e., 2008, 2009 and 2011, to investigate whether our previous results might be influenced by crisis periods. Indeed, the 2007 financial crisis and the 2011 European Sovereign Debt Crisis hampered access to bank credit (Kayshap and Stein 2000; Puri et al. 2011; Jiménez et al. 2012; Jiménez et al. 2014) with a disproportionately greater effect for private, more informationally opaque firms (De Young et al. 2015). The results, shown in Table 5, confirm not only the influence of the trade credit channel on firm investment decisions (column (1)) but also the significant moderating effects of cooperative banking (column (2)) and relationship banking (columns (3) and (4)).

**Table 5:** Robustness: trade credit in off-crisis periods

Variables	(1)	(2)	(3)	(4)
	FE panel model	FE panel model	FE panel model	FE panel model
NTC	− 0.267***	− 0.428***	− 0.356***	− 0.248*
	(0.089)	(0.124)	(0.119)	(0.127)
NTC * cooperative banking		0.260**		
		(0.143)		
Cooperative banking		0.002		
		(0.027)		
NTC * length of relationship			0.010*	
			(0.006)	
Length of relationship			0.022***	
			(0.008)	
NTC * borrower-to-branch distance				− 0.058
				(0.147)
Borrower-to-branch distance				− 0.327
				(0.314)
ULC	− 0.007***	− 0.007***	− 0.007***	− 0.007***
	(0.001)	(0.001)	(0.001)	(0.001)
Bank debt/total liabilities	− 0.001	− 0.001	− 0.001	− 0.001
	(0.001)	(0.001)	(0.001)	(0.001)
Financial fixed/total assets	0.985***	0.990***	0.967***	1.007***
	(0.215)	(0.214)	(0.215)	(0.217)
Intangible assets/total assets	0.024***	0.024***	0.024***	0.024***
	(0.003)	(0.003)	(0.003)	(0.003)
Tangible assets/total assets	0.012***	0.012***	0.012***	0.011***
	(0.001)	(0.001)	(0.001)	(0.001)
Inventory/total assets	− 0.003**	− 0.003**	− 0.003**	− 0.003**
	(0.001)	(0.001)	(0.001)	(0.001)
ROA	0.000	0.000	0.000	0.001
	(0.002)	(0.002)	(0.002)	(0.002)
Observations	10,804	10,733	10,698	10,264
R-squared	0.14	0.14	0.14	0.14
Number of firm-bank pairs	5866	5828	5817	5550
Yearly FE	No	No	No	No
Industry FE	No	No	No	No
Year × industry FE	Yes	Yes	Yes	Yes

The table presents the results of the FE panel regression analysis for the Trade Credit in Off-Crisis Periods robustness models of Eqs. (1), (2), and (3) where the dependent variable is $\Delta CAPEX_{i,k,n,t}$ which is the annual percentage change in the firm’s capital expenditure. $NTC_{i,k,n,t}$ represents the trade credit channel measured as the difference between the firm’s account receivables and account payables (i.e., net trade credit) scaled by the firm’s size as measured by total assets. All variables are defined in Table 1. Year, Industry and Year*Industry fixed effects are incorporated in regressions where indicated (not reported). Robust errors, reported in parentheses, are clustered at the bank-firm (pair) level

***p < 0.01, **p < 0.05, *p < 0.1, respectively

Secondly, we aim to conduct the analysis on the period of financial instability to investigate whether the equilibrium in the relationship between trade credit channel and firm investment switches in normal and crisis periods. To capture the effect of crisis periods, we constructed a time dummy $d_{crisis}$ that allows us to capture the specific effect of crisis periods which might affect our main results, and interact the latter with our regressor of interest $NTC$, giving rise to $NTC *d_{crisis}$, to examine whether the effect of net trade credit on firm investment significantly changes during crisis periods. The estimation results of our baseline model with the crisis effect are reported in Table 6. In the interaction analysis, the influence of the trade credit channel now also depends on the value of the time dummy $d_{crisis}$. When its value is 0, i.e., in normal economic conditions, the effect of the lower-order term $NTC$ is negative and statistically significant, corroborating our previous baseline model’s results in Eq. (1). However, when its value is 1, i.e., during the financial crisis, the effect of the term $NTC$ is given by adding the coefficients of the lower-order term $NTC$ to the one of the interaction term $NTC *d_{crisis}$ that turns out to be negative and statistically significant (column (1)). Therefore, even if trade credit as a funding source still positively affects firm investment behavior, we argue that this beneficial effect is increased in periods of financial distress. We label this novel evidence as the “crisis effect”. The interpretation of this result suggests that, during crisis years, the importance of the trade channel as a financing tool increases to compensate for the reduced supply of bank loans in a scenario where firms have to search for alternative financing sources. As a consequence, SMEs’ reliance on the trade credit channel to finance their investments increases in a setting where they are even more credit-constrained, in line with our previous results. Interestingly, the presence of cooperative banks and longer firm-bank relationships weaken firm reliance on trade credit to spur investments even more during crises periods, as documented by the positive and significant coefficients on the triple interaction terms NTC * $d_{crisis}$ * cooperative banking (column (2)) and NTC * $d_{crisis}$ * length of relationship (column (3)), further corroborating our main empirical findings.

**Table 6:** Robustness: trade Credit with the crisis effect

Variables	(1)	(2)	(3)	(4)
	FE panel model	FE panel model	FE panel model	FE panel model
NTC	− 0.356***	− 0.451***	− 0.395***	− 0.432***
	(0.105)	(0.108)	(0.130)	(0.104)
NTC * $d_{crisis}$	− 0.092***	− 0.092**	− 0.129**	− 0.101***
	(0.030)	(0.043)	(0.049)	(0.037)
$d_{crisis}$	− 0.049***	− 0.043*	0.025	1.033***
	(0.014)	(0.029)	(0.017)	(0.035)
NTC * $d_{crisis}$ * cooperative banking		0.032**		
		(0.015)		
Cooperative banking * $d_{crisis}$		− 0.012		
		(0.019)		
NTC * cooperative banking		0.157*		
		(0.090)		
Cooperative banking		0.009		
		(0.032)		
NTC * $d_{crisis}$ * length of relationship			0.006*	
			(0.003)	
Length of relationship * $d_{crisis}$			0.003***	
			(0.001)	
NTC * length of relationship			0.005*	
			(0.006)	
Length of relationship			0.144***	
			(0.006)	
NTC * $d_{crisis}$ * borrower-to-branch distance				0.015
				(0.045)
Borrower-to-branch distance * $d_{crisis}$				− 0.025*
				(0.015)
NTC * borrower-to-branch distance				0.108
				(0.114)
Borrower-to-branch distance				
ULC	− 0.005***	− 0.005***	− 0.005***	− 0.005***
	(0.001)	(0.001)	(0.001)	(0.001)
Bank debt/total liabilities	− 0.000	− 0.000	− 0.000	− 0.000
	(0.001)	(0.001)	(0.001)	(0.001)
Financial fixed/total assets	1.214***	1.215***	1.209***	1.206***
	(0.191)	(0.190)	(0.194)	(0.194)
Intangible assets/total assets	0.022***	0.022***	0.022***	0.022***
	(0.002)	(0.002)	(0.002)	(0.002)
Tangible assets/total assets	0.014***	0.014***	0.014***	0.013***
	(0.001)	(0.001)	(0.001)	(0.001)
Inventory/total assets	− 0.004***	− 0.004***	− 0.004***	− 0.004***
	(0.001)	(0.001)	(0.001)	(0.001)
ROA	0.005**	0.005**	0.004**	0.005**
	(0.002)	(0.002)	(0.002)	(0.002)
Observations	16,328	16,224	16,166	15,576
R-squared	0.16	0.16	0.16	0.16
Number of firm-bank pairs	6550	6509	6499	6220
Yearly FE	No	No	No	No
Industry FE	No	No	No	No
Year × industry FE	Yes	Yes	Yes	Yes

The table presents the results of the FE panel regression analysis for the Trade Credit with the Crisis Effect robustness models of Eqs. (1), (2), and (3) where the dependent variable is $\Delta CAPEX_{i,k,n,t}$ which is the annual percentage change in the firm’s capital expenditure. $NTC_{i,k,n,t}$ represents the trade credit channel measured as the difference between the firm’s account receivables and account payables (i.e., net trade credit) scaled by the firm’s size as measured by total assets. All variables are defined in Table 1. Year, Industry and Year*Industry fixed effects are incorporated in regressions where indicated (not reported). Robust errors reported in parentheses are clustered at the industry level

***p < 0.01, **p < 0.05, *p < 0.1, respectively

### Accounting for firm heterogeneity

We now verify whether our results might differ across firms of different collateralization levels since collateral plays an important role in the firm capability of raising external funding. In this regard, empirical evidence shows that higher-collateralized firms face a lower cost of debt and benefit from higher availability of external finance (Benmelech and Bergman 2011). Thus, firms which are more collateralized are less likely to be financially constrained, thus affecting their reliance on the trade credit channel as a funding source. For this purpose, we created the binary variable *high collateral* that takes a value of 1 for those firms whose ratio of fixed tangible assets over total assets is above the median value of the distribution and 0 otherwise. The results are displayed in Table 7. As expected, firm reliance on the trade credit channel to boost investments is decreasing in the degree of firm collateralization due to increased access to bank credit, as documented by the positive and statistically coefficient associated with the interaction term NTC*high collateral reported in column (1). Indeed, Cerqueiro et al. (2016) show that collateral plays an important and positive role in the provision of lending, while Benmelech and Bergman (2011) find a negative relationship between collateral and the cost of external debt finance. Moreover, greater collateral strengthens the moderating effects of cooperative banking and relationship banking on the nexus between trade credit and capital investment, as documented by the positive and significant coefficients on the triple interaction terms NTC * high Collateral * cooperative banking (column (2)) and NTC * high Collateral * length of relationship (column (3)).

**Table 7:** Robustness: low versus high collateralized firms

Variables	(1)	(2)	(3)	(4)
	FE panel model	FE panel model	FE panel model	FE panel model
NTC	− 0.431***	− 0.578***	− 0.510***	− 0.622***
	(0.073)	(0.089)	(0.097)	(0.107)
Cooperative banking		0.030		
		(0.032)		
High collateral	0.049**	0.074**	0.060*	0.016
	(0.023)	(0.032)	(0.032)	(0.036)
NTC * cooperative banking		0.269***		
		(0.098)		
NTC * high collateral	0.120*	0.258**	0.153	0.399***
	(0.072)	(0.107)	(0.108)	(0.120)
High collateral * cooperative banking		0.048		
		(0.035)		
NTC * high collateral * cooperative banking		0.261**		
		(0.131)		
Length of relationship			0.009	
			(0.006)	
NTC * length of relationship			0.011	
			(0.007)	
Length of relationship * high collateral			0.001	
			(0.002)	
NTC * high collateral * length of relationship			0.007*	
			(0.004)	
NTC * borrower-to-branch distance				0.264
				(0.226)
Borrower-to-branch distance				− 0.257
				(0.213)
Borrower-to-branch distance * high collateral				0.046
				(0.044)
NTC * high collateral * borrower-to-branch distance				0.401
				(0.353)
ULC	− 0.005***	− 0.005***	− 0.005***	− 0.005***
	(0.001)	(0.001)	(0.001)	(0.001)
Bank debt/total liabilities	− 0.000	− 0.000	− 0.000	− 0.000
	(0.001)	(0.001)	(0.001)	(0.001)
Financial fixed/total assets	1.222***	1.235***	1.215***	1.221***
	(0.157)	(0.156)	(0.156)	(0.163)
Intangible assets/total assets	0.021***	0.021***	0.021***	0.022***
	(0.002)	(0.002)	(0.002)	(0.002)
Tangible assets/total assets	0.012***	0.012***	0.012***	0.012***
	(0.001)	(0.001)	(0.001)	(0.001)
Inventory/total assets	− 0.004***	− 0.004***	− 0.004***	− 0.004***
	(0.001)	(0.001)	(0.001)	(0.001)
ROA	0.005***	0.004***	0.004***	0.005***
	(0.001)	(0.001)	(0.001)	(0.001)
Observations	16,328	16,224	16,166	15,576
R-squared	0.16	0.16	0.16	0.16
Number of firm-bank pairs	6550	6509	6499	6220
Yearly FE	No	No	No	No
Industry FE	No	No	No	No
Year × industry FE	Yes	Yes	Yes	Yes

The table presents the results of the FE panel regression analysis for the Low versus High Collateralised Firms robustness models of Eqs. (1), (2), and (3) where the dependent variable is $\Delta CAPEX_{i,k,n,t}$ which is the annual percentage change in the firm’s capital expenditure. $NTC_{i,k,n,t}$ represents the trade credit channel measured as the difference between the firm’s account receivables and account payables (i.e., net trade credit) scaled by the firm’s size as measured by total assets. The binary variable *high collateral* takes the value of 1 for those firms whose ratio of fixed tangible assets over total assets is above the median value of the distribution and 0 otherwise. All variables are defined in Table 1. Year, Industry and Year*Industry fixed effects are incorporated in regressions where indicated (not reported). Robust errors, reported in parentheses, are clustered at the bank-firm (pair) level

***p < 0.01, **p < 0.05, *p < 0.1, respectively

### Longer trade credit period

As an additional robustness test, due to the short-term nature of trade credit contracts, we now test whether our results might be driven by those firms characterized by a trade credit period exceeding 365 days. To this purpose, we now perform our empirical analysis by removing from the analysis SMEs having a trade credit period exceeding one year with both days sales (DSO) and days payable (DPO) outstanding over 365 days. The results, displayed in Table 8, leave our main results unaffected.

**Table 8:** Robustness: trade credit period

Variables	(1)	(2)	(3)	(4)
	FE panel model	FE panel model	FE panel model	FE panel model
NTC	− 0.393***	− 0.513***	− 0.462***	− 0.467***
	(0.068)	(0.086)	(0.092)	(0.100)
NTC * cooperative banking		0.208**		
		(0.087)		
Cooperative banking		− 0.013		
		(0.027)		
NTC * length of relationship			0.008**	
			(0.004)	
Length of relationship			0.316**	
			(0.142)	
NTC * borrower-to-branch distance				0.096
				(0.115)
Borrower-to-branch distance				− 0.327
				(0.314)
ULC	− 0.006***	− 0.006***	− 0.006***	− 0.006***
	(0.002)	(0.002)	(0.002)	(0.002)
Bank debt/total liabilities	− 0.000	− 0.000	− 0.000	− 0.000
	(0.001)	(0.001)	(0.001)	(0.001)
Financial fixed/total assets	1.250***	1.250***	1.243***	1.231***
	(0.166)	(0.164)	(0.165)	(0.171)
Intangible assets/total assets	0.022***	0.022***	0.022***	0.022***
	(0.002)	(0.002)	(0.002)	(0.002)
Tangible assets/total assets	0.014***	0.014***	0.014***	0.013***
	(0.001)	(0.001)	(0.001)	(0.001)
Inventory/total assets	− 0.004***	− 0.004***	− 0.004***	− 0.004***
	(0.001)	(0.001)	(0.001)	(0.001)
ROA	0.004***	0.004***	0.004***	0.004***
	(0.001)	(0.001)	(0.001)	(0.001)
Observations	15,211	15,109	15,063	14,499
R-squared	0.15	0.16	0.16	0.16
Number of firm-bank pairs	6175	6135	6128	5859
Yearly FE	No	No	No	No
Industry FE	No	No	No	No
Year × industry FE	Yes	Yes	Yes	Yes

The table presents the results of the FE panel regression analysis for the Trade Credit Period robustness models of Eqs. (1), (2), and (3) where the dependent variable is $\Delta CAPEX_{i,k,n,t}$ which is the annual percentage change in the firm’s capital expenditure. $NTC_{i,k,n,t}$ represents the trade credit channel measured as the difference between the firm’s account receivables and account payables (i.e., net trade credit) scaled by the firm’s size as measured by total assets. All variables are defined in Table 1. Year, Industry and Year*Industry fixed effects are incorporated in regressions where indicated (not reported). Robust errors, reported in parentheses, are clustered at the bank-firm (pair) level

***p < 0.01, **p < 0.05, *p < 0.1, respectively

### Addressing potential endogeneity concerns

In this section we address the endogeneity associated with potential reverse causality issues and omitted variable bias resulting from the simultaneous specification of the trade credit and firm investment variables. Therefore, to assuage concerns about potential endogeneity issues that might affect our estimation results, we perform instrumental variable (IV) estimation with respect to our models of Eqs. (1), (2), and (3) by using banking-related instruments. Firstly, we use as instrumental variables the equity ratio *(Equity Ratio),* the non-performing loan ratio *(NPL ratio),* the return on average assets *(ROAA)* and the bank size expressed as the logarithm of total assets *(Bank Size)*, following Storz et al. (2017), Ferrando and Wolski (2018) and Norden et al. (2020). In this regard, we follow the argument that the situation of a financing bank should be unrelated to company’s investment decisions but may affect the degree of trade credit in the corporate sector (Ferrando and Wolski 2018),^14^ assuming that banks are not active firms’ investors playing a determinant role in the firm’s operating and financing decisions. Therefore, these instruments could be referred to as pure numbers that are likely to affect the firm net trade credit position without directly affecting our dependent variable represented by the annual percentage change in the firm’s capital expenditure. The validity and importance of the instruments for the control variables are verified using a number of diagnostic tests reported at the bottom of Table 9, which reports the second stage of the regressions and the value of the coefficients and standard errors of the instrumental variables in the first stage. Results are reported in Table 9. Our variable of interest is the firm net trade credit position. Consistent with our previous results shown in Table 2, instrumental variable estimation confirms that an increase in net trade credit negatively affects firm investment behavior and that this effect is statistically significant at the 1% level (column (1)). Furthermore, IV estimation further corroborates that cooperative banking and relationship banking significantly moderate the nexus between trade credit and capital investment, in line with our main results. Overall, the results obtained by performing IV estimation are therefore in line with our main empirical findings and the diagnostic tests do not specify any problems regarding the application of the instruments used, thus providing a reliable robustness check for our main results.

**Table 9:** Robustness: IV estimation

Variables	(1)	(1)	(2)	(3)
	IV estimation	IV estimation	IV estimation	IV estimation
NTC	− 8.623***	− 4.806*	− 2.894*	− 4.888*
	(1.346)	(1.238)	(0.272)	(1.128)
NTC * cooperative banking		3.455*		
		(1.152)		
Cooperative banking		− 0.387		
		(0.768)		
NTC * length of relationship			0.077*	
			(0.213)	
Length of relationship			0.009	
			(0.022)	
NTC * borrower-to-branch distance				5.887
				(15.455)
Borrower-to-branch distance				− 0.327
				(0.317)
Observations	15,438	12,561	12,520	11,968
Number of firm-bank pairs	5128	3994	3977	3755
Tests				
Kleibergen-Paap	0.0000	0.0773	0.0211	0.0862
Anderson-Rubin	0.0000	0.0000	0.0209	0.0000
Stock-Wright	0.0000	0.0000	0.0206	0.0000
Hansen J	0.5248	0.6709	0.3690	0.7155
*First stage*				
Equity ratio	0.005***	0.005***	0.005***	0.0055***
	(0.001)	(0.001)	(0.001)	(0.001)
NPL ratio	0.002***	0.002***	0.001***	0.0018***
	(0.001)	(0.001)	(0.001)	(0.001)
ROAA	− 0.001	− 0.0013	− 0.001	− 0.0015
	(0.001)	.0013	(0.0013)	(0.001)
Bank size	0.055***	0.049***	0.008***	0.0544***
	(0.009)	(0.009)	(0.0135)	(0.010)

The table reports in columns (1)–(4) coefficient estimates and robust standard errors (in parentheses) for the two-stage treatment effects robustness models of Eqs. (1), (2), and (3). We treat NTC as endogenous and we use banking-related instruments. The sample period is 2008–2014. The first stage includes all explanatory variables in the second stage. The dependent variable is $\Delta CAPEX_{i,k,n,t}$ which is the annual percentage change in the firm’s capital expenditure. $NTC_{i,k,n,t}$ represents the trade credit channel measured as the difference between the firm’s account receivables and account payables (i.e., net trade credit) scaled by the firm’s size as measured by total assets. The Kleibergen-Paap is a test of under-identification distributed as chi-square under the null of under-identification. The Anderson Rubin and Stock-Wright LM S statistic are weak-instrument-robust inference tests, distributed as F-test and chi-square respectively, under the null that coefficients of the endogenous regressors in the structural equation are jointly equal to zero, and the over-identifying restrictions are valid. The Hansen J statistic is a test of the over-identifying restrictions, distributed as chi-square under the null of instrument validity. The first-stage Kleibergen-Paap rk Wald F statistic is a test for weak instrument. All variables are defined in Table 1. In the margin, we report coefficients and standard errors for the instrumental variables. Robust errors, reported in parentheses, are clustered at the bank-firm (pair) level

***p < 0.01, **p < 0.05, *p < 0.1, respectively

### Additional controls

To control for the possible influence of extra firm-specific shocks on our estimation results, we now run the main model specifications by controlling for the borrowing firm’s regional fixed effects to control for possible macroeconomic shocks at the regional or provincial level which may affect firm investment decisions other than the trade credit channel. The results are displayed in Table 10. Our main findings remain, even in this case, qualitatively unchanged.^15^

**Table 10:** Robustness: regional fixed effects

Variables	(1)	(2)	(3)	(4)
	FE panel model	FE panel model	FE panel model	FE panel model
NTC	− 0.390***	− 0.499***	− 0.460***	− 0.474***
	(0.066)	(0.080)	(0.087)	(0.095)
NTC * cooperative banking		0.193**		
		(0.080)		
Cooperative banking		− 0.001		
		(0.025)		
NTC * length of relationship			0.009*	
			(0.006)	
Length of relationship			0.009*	
			(0.005)	
NTC * borrower-to-branch distance				0.117
				(0.109)
Borrower-to-branch distance				− 0.257
				(0.213)
ULC	− 0.005***	− 0.005***	− 0.005***	− 0.005***
	(0.001)	(0.001)	(0.001)	(0.001)
Bank debt/total liabilities	− 0.000	− 0.000	− 0.000	− 0.000
	(0.001)	(0.001)	(0.001)	(0.001)
Financial fixed/total assets	1.210***	1.218***	1.200***	1.210***
	(0.157)	(0.156)	(0.156)	(0.161)
Intangible assets/total assets	0.022***	0.022***	0.022***	0.022***
	(0.002)	(0.002)	(0.002)	(0.002)
Tangible assets/total assets	0.014***	0.014***	0.014***	0.013***
	(0.001)	(0.001)	(0.001)	(0.001)
Inventory/total assets	− 0.004***	− 0.004***	− 0.004***	− 0.004***
	(0.001)	(0.001)	(0.001)	(0.001)
ROA	0.005***	0.005***	0.004***	0.005***
	(0.001)	(0.001)	(0.001)	(0.001)
Observations	16,224	16,224	16,062	15,576
R-squared	0.16	0.16	0.16	0.16
Number of firm-bank pairs	6509	6509	6458	6220
Yearly FE	No	No	No	No
Industry FE	No	No	No	No
Year × industry FE	Yes	Yes	Yes	Yes
Regional FE	Yes	Yes	Yes	Yes

The table presents the results of the FE panel regression analysis for the Regional Fixed Effects robustness models of Eqs. (1), (2), and (3) where the dependent variable is $\Delta CAPEX_{i,k,n,t}$ which is the annual percentage change in the firm’s capital expenditure. $NTC_{i,k,n,t}$ represents the trade credit channel measured as the difference between the firm’s account receivables and account payables (i.e., net trade credit) scaled by the firm’s size as measured by total assets. All variables are defined in Table 1. Year, Industry and Year*Industry fixed effects are incorporated in regressions where indicated (not reported). Robust errors, reported in parentheses, are clustered at the bank-firm (pair) level

***p < 0.01, **p < 0.05, *p < 0.1, respectively

### Commercial banks and Italian SMEs

To account for the fact that commercial (non-cooperative) banks make up for the other 92.8% of the Italian loan market (McKillop et. al. 2020) and have an important role not only in financing large firms, but also in financing Italian SMEs (e.g., Bronzini and D’Ignazio (2017) show that international banks facilitate exports of Italian SMEs), we now control for the proportion of non-cooperative banks’ branches located in a given province with respect to the number of firms operating in that province, i.e., *commercial banking*. Indeed, we now control for the provincial presence of other non-cooperative banks’ branches to rule out issues related to ignoring large commercial banks (using a more arms-length, i.e., hard information-based, type of lending) acting as SME financiers. This analysis permits to disentangle between the “general funding” story from the “specific soft information-based lending funding” story. Indeed, controlling for how the presence of large commercial banks overlaps with the presence of cooperative banks by Italian province provides further confirmatory evidence that the density of cooperative banks per firm reflects how much SMEs effectively fund from the latter and less from the former. Specifically, we repeat the analyses of Tables 2, 3, and 4 as per Eqs. (1), (2), and (3), respectively, while controlling for *commercial banking*. The results, presented in Table 11, once again leave our main findings qualitatively and quantitatively unchanged.

**Table 11:** Robustness: commercial banking

Variables	(1)	(2)	(3)	(4)
	FE panel model	FE panel model	FE panel model	FE panel model
NTC	− 0.390***	− 0.499***	− 0.460***	− 0.474***
	(0.066)	(0.080)	(0.087)	(0.094)
NTC * cooperative banking		0.193***		
		(0.080)		
Cooperative banking		− 0.001		
		(0.025)		
NTC * length of relationship			0.009*	
			(0.006)	
Length of relationship			0.005	
			(0.015)	
NTC * borrower-to-branch distance				0.117
				(0.109)
Borrower-to-branch distance				− 0.255
				(0.211)
Commercial banking	− 0.006	− 0.003	− 0.004	− 0.001
	(0.014)	(0.014)	(0.014)	(0.015)
ULC	− 0.005***	− 0.005***	− 0.005***	− 0.005***
	(0.001)	(0.001)	(0.001)	(0.001)
Bank debt/total liabilities	− 0.000	− 0.000	− 0.000	− 0.000
	(0.001)	(0.001)	(0.001)	(0.001)
Financial fixed/total assets	1.210***	1.219***	1.200***	1.210***
	(0.157)	(0.156)	(0.156)	(0.161)
Intangible assets/total assets	0.022***	0.022***	0.022***	0.022***
	(0.002)	(0.002)	(0.002)	(0.002)
Tangible assets/total assets	0.014***	0.014***	0.014***	0.013***
	(0.001)	(0.001)	(0.001)	(0.001)
Inventory/total assets	− 0.004***	− 0.004***	− 0.004***	− 0.004***
	(0.001)	(0.001)	(0.001)	(0.001)
ROA	0.005***	0.005***	0.004***	0.005***
	(0.001)	(0.001)	(0.001)	(0.001)
Observations	16,224	16,224	16,062	15,576
Number of firm-bank pairs	6509	6509	6458	6220
R-squared	0.16	0.16	0.16	0.16
Yearly FE	No	No	No	No
Industry FE	No	No	No	No
Year × industry FE	Yes	Yes	Yes	Yes

The table presents the results of the FE panel regression analysis for the Commercial Banking robustness models of Eqs. (1), (2), and (3) where the dependent variable is $\Delta CAPEX_{i,k,n,t}$ which is the annual percentage change in the firm’s capital expenditure. $NTC_{i,k,n,t}$ represents the trade credit channel measured as the difference between the firm’s account receivables and account payables (i.e., net trade credit) scaled by the firm’s size as measured by total assets. All variables are defined in Table 1. Year, Industry and Year*Industry fixed effects are incorporated in regressions where indicated (not reported). Robust errors, reported in parentheses, are clustered at the bank-firm (pair) level

***p < 0.01, **p < 0.05, *p < 0.1, respectively

### Disentangling uptaken and provided trade credit

We now exploit more of the variation in the trade credit channel by separately looking into trade credit provided to clients and trade credit uptaken from suppliers. Specifically, we now run our models of Eqs. (1), (2), and (3) by splitting between *uptaken* (i.e., payables/total assets) and *provided* (i.e., receivables/total assets) *trade credit*. The results, presented in Table 12, not only confirm our first hypothesis (H1) by showing a negative and statistically significant effect on firm investment of trade credit provided to customers, and a positive and statistically significant effect of trade credit uptaken from suppliers (column (1)), but also validate our second and third hypotheses (H2 and H3) as cooperative banking and relationship banking weakens the influence of the trade credit channel on firm investment decisions (columns (2) and (3)).

**Table 12:** Robustness: uptaken and provided trade credit

Variables	(1)	(2)	(3)	(4)
	FE panel model	FE panel model	FE panel model	FE panel model
Provided trade credit	− 0.165*	− 0.281***	− 0.235*	− 0.247**
	(0.097)	(0.109)	(0.120)	(0.123)
Uptaken trade credit	0.536***	0.622***	0.607***	0.655***
	(0.083)	(0.102)	(0.113)	(0.123)
Provided trade credit * cooperative banking		0.213**		
		(0.088)		
Uptaken trade credit * cooperative banking		− 0.157**		
		(0. 080)		
Cooperative banking		− 0.019		
		(0.047)		
Provided trade credit * length of relationship			0.009*	
			(0.005)	
Uptaken trade credit *length of relationship			− 0.009*	
			(0.005)	
Length of relationship			0.007	
			(0.007)	
Provided trade credit * borrower-to-branch distance				0.100
				(0.133)
Uptaken trade credit * borrower-to-branch distance				− 0.178
				(0.146)
Borrower-to-branch distance				− 0.258
				(0.214)
ULC	− 0.005***	− 0.005***	− 0.005***	− 0.005***
	(0.001)	(0.001)	(0.001)	(0.001)
Bank debt/total liabilities	0.000	0.000	0.000	0.000
	(0.001)	(0.001)	(0.001)	(0.001)
Financial fixed/total assets	1.410***	1.418***	1.404***	1.397***
	(0.166)	(0.166)	(0.166)	(0.170)
Intangible assets/total assets	0.024***	0.024***	0.024***	0.024***
	(0.003)	(0.003)	(0.003)	(0.003)
Tangible assets/total assets	0.015***	0.015***	0.016***	0.015***
	(0.001)	(0.001)	(0.001)	(0.001)
Inventory/total assets	− 0.002**	− 0.002**	− 0.002*	− 0.003**
	(0.001)	(0.001)	(0.001)	(0.001)
ROA	0.005***	0.005***	0.005***	0.006***
	(0.001)	(0.001)	(0.001)	(0.001)
Observations	16,328	16,224	16,166	15,576
R-squared	0.16	0.16	0.16	0.16
Number of firm-bank pairs	6550	6509	6499	6220
Yearly FE	No	No	No	No
Industry FE	No	No	No	No
Year x industry FE	Yes	Yes	Yes	Yes

The table presents the results of the FE panel regression analysis for the Uptaken and Provided Trade Credit robustness models of Eqs. (1), (2), and (3) where the dependent variable is $\Delta CAPEX_{i,k,n,t}$ which is the annual percentage change in the firm’s capital expenditure. Uptaken Trade Credit is measured as the firm’s payables over total assets, whereas Provided Trade Credit is computed as the firm’s receivables over total assets. All variables are defined in Table 1. Year, Industry and Year*Industry fixed effects are incorporated in regressions where indicated (not reported). Robust errors, reported in parentheses, are clustered at the bank-firm (pair) level

***p < 0.01, **p < 0.05, *p < 0.1, respectively

### Alternative proxies to measure trade credit

To mitigate concerns related to the fact that our measure of trade credit, defined as the net effect of extended and received trade credit scaled by total assets, shows the liquidity burden to the firm, rather than depicting trade credit as a funding resource for the firm, we now test our hypotheses (H1), (H2), and (H3) according to Eqs. (1), (2), and (3), respectively, by using alternative proxies for trade credit drawing from the extant literature.

Firstly, following Carbo-Valverde et al. (2016) who use the Account Payables-to-Total Liabilities ratio to measure trade credit, we repeat the analysis and test our hypotheses (H1), (H2), and (H3) according to models (1), (2), and (3), respectively, by using the ratio Account Payable-to-Total Liabilities ratio as a further proxy for trade credit. The results are shown in Table 13.

**Table 13:** Robustness: alternative measure of trade credit: account payables-to-total liabilities ratio

Variables	(1)	(2)	(3)	(4)	(5)	(6)	(7)	(8)
	FE panel model	FE panel model	FE panel model	FE panel model	FE panel model	FE panel model	FE panel model	FE panel model
Account payables-to-total liabilities	0.537***	0.530***	0.596***	0.589***	0.590***	0.589***	0.642***	0.645***
	(0.083)	(0.083)	(0.101)	(0.102)	(0.112)	(0.112)	(0.126)	(0.123)
Account payables-to-total liabilities * cooperative banking			− 0.110	− 0.109				
			(0.104)	(0.105)				
Cooperative banking			0.055	0.053				
			(0.035)	(0.036)				
Account payables-to-total liabilities * length of relationship					− 0.007	− 0.008		
					(0.008)	(0.008)		
Length of relationship					− 0.115***	0.017***		
					(0.025)	(0.004)		
Account payables-to-total liabilities * borrower-to-branch distance							− 0.152	− 0.171
							(0.148)	(0.145)
Borrower-to-branch distance							− 0.257	− 0.327
							(0.213)	(0.314)
Controls	Yes	Yes	Yes	Yes	Yes	Yes	Yes	Yes
Observations	16,335	16,335	16,231	16,231	16,172	16,172	15,583	15,583
R-squared	0.11	0.16	0.11	0.16	0.11	0.16	0.11	0.16
Number of firm-bank pairs	6553	6553	6512	6512	6501	6501	6223	6223
Yearly FE	Yes	No	Yes	No	Yes	No	Yes	No
Industry FE	Yes	No	Yes	No	Yes	No	Yes	No
Year × industry FE	No	Yes	No	Yes	No	Yes	No	Yes

The table presents the results of the FE panel regression analysis for the Account Payables-to-Total Liabilities robustness models of Eqs. (1), (2), and (3) where the dependent variable is $\Delta CAPEX_{i,k,n,t}$ which is the annual percentage change in the firm’s capital expenditure. $Account\;Payables - to - Total\;Liabilities_{i,k,n,t}$ represents an alternative measure for the trade credit channel. All variables are defined in Table 1. Year, Industry and Year*Industry fixed effects are incorporated in regressions where indicated (not reported). Robust errors, reported in parentheses, are clustered at the bank-firm (pair) level

***p < 0.01, **p < 0.05, *p < 0.1, respectively

Secondly, following Love et al. (2007), Goncalves et al. (2018), and D'Mello and Toscano (2020), we measure trade credit as the Account Payables-to-Costs of Goods Sold ratio to assuage the concern that trade credit, as a funding source, should be reflected more directly by the account payables, rather than receivables. The results are displayed in Table 14.

**Table 14:** Robustness: alternative measure of trade credit account payables-to-costs of goods sold

Variables	(1)	(2)	(3)	(4)	(5)	(6)	(7)	(8)
	FE panel model	FE panel model	FE panel model	FE panel model	FE panel model	FE panel model	FE panel model	FE panel model
Account payables-to-costs of goods sold	0.158***	0.169***	0.108**	0.120***	0.185***	0.198***	0.166***	0.177***
	(0.034)	(0.033)	(0.042)	(0.042)	(0.053)	(0.052)	(0.050)	(0.050)
Account payables-to-costs of goods sold * cooperative banking			0.098**	0.095**				
			(0.049)	(0.049)				
Cooperative banking			− 0.013	− 0.014				
			(0.029)	(0.030)				
Account payables-to-costs of goods sold * length of relationship					− 0.003	− 0.003		
					(0.004)	(0.004)		
Length of relationship					− 0.121***	− 0.024*		
					(0.025)	(0.013)		
Account payables-to-costs of goods sold * borrower-to-branch distance							− 0.018	− 0.019
							(0.066)	(0.064)
Borrower-to-branch distance							− 0.259	− 0.329
							(0.215)	(0.316)
Controls	Yes	Yes	Yes	Yes	Yes	Yes	Yes	Yes
Observations	16,335	16,335	16,231	16,231	16,172	16,172	15,583	15,583
R-squared	0.10	0.15	0.10	0.16	0.10	0.16	0.10	0.16
Number of firm-bank pairs	6553	6553	6512	6512	6501	6501	6223	6223
Yearly FE	Yes	No	Yes	No	Yes	No	Yes	No
Industry FE	Yes	No	Yes	No	Yes	No	Yes	No
Year × industry FE	No	Yes	No	Yes	No	Yes	No	Yes

The table presents the results of the FE panel regression analysis for the Account Payables-to-Costs of Goods Sold robustness models of Eqs. (1), (2), and (3) where the dependent variable is $\Delta CAPEX_{i,k,n,t}$ which is the annual percentage change in the firm’s capital expenditure. $Account\;Payables - to - Costs\;of\;Goods\;Sold_{i,k,n,t}$ represents an alternative measure for the trade credit channel. All variables are defined in Table 1. Year, Industry and Year*Industry fixed effects are incorporated in regressions where indicated (not reported). Robust errors, reported in parentheses, are clustered at the bank-firm (pair) level

***p < 0.01, **p < 0.05, *p < 0.1, respectively

Lastly, we test the robustness of our results by scaling the net effect of extended and received trade credit, i.e., account payables minus account receivables, by total sales instead of total assets, consistent with Love et al. (2007), Goncalves et al. (2018), and D'Mello and Toscano (2020). The results are presented in Table 15.

**Table 15:** Robustness: alternative measure of trade credit, NTC scaled by total sales

Variables	(1)	(2)	(3)	(4)	(5)	(6)	(7)	(8)
	FE panel model	FE panel model	FE panel model	FE panel model	FE panel model	FE panel model	FE panel model	FE panel model
(Account payables—account receivables)/total sales	− 0.131***	− 0.129***	− 0.187***	− 0.186***	− 0.193***	− 0.180***	− 0.173**	− 0.155**
	(0.043)	(0.042)	(0.054)	(0.052)	(0.064)	(0.061)	(0.075)	(0.068)
(Account payables—account receivables)/total sales * cooperative banking			0.105*	0.107*				
			(0.062)	(0.060)				
Cooperative banking			0.011	0.009				
			(0.025)	(0.024)				
(Account payables—account receivables)/total sales * length of relationship					0.008*	0.006*		
					(0.004)	(0.004)		
Length of relationship					− 0.121***	0.034**		
					(0.025)	(0.015)		
(Account payables—account receivables)/total sales * borrower-to-branch distance							0.060	0.040
							(0.083)	(0.079)
Borrower-to-branch distance							− 0.249	− 0.319
							(0.203)	(0.306)
Controls	Yes	Yes	Yes	Yes	Yes	Yes	Yes	Yes
Observations	16,312	16,312	16,208	16,208	16,150	16,150	15,560	15,560
R-squared	0.10	0.15	0.10	0.15	0.10	0.15	0.10	0.15
Number of firm-bank pairs	6541	6541	6500	6500	6490	6490	6211	6211
Yearly FE	Yes	No	Yes	No	Yes	No	Yes	No
Industry FE	Yes	No	Yes	No	Yes	No	Yes	No
Year × industry FE	No	Yes	No	Yes	No	Yes	No	Yes

The table presents the results of the FE panel regression analysis for the NTC Scaled by Total Sales robustness models of Eqs. (1), (2), and (3) where the dependent variable is $\Delta CAPEX_{i,k,n,t}$ which is the annual percentage change in the firm’s capital expenditure. $\left( {Account\;Payables - Account\;Receivables} \right)/Total\;Sales_{i,k,n,t}$ represents an alternative measure for the trade credit channel. All variables are defined in Table 1. Year, Industry and Year*Industry fixed effects are incorporated in regressions where indicated (not reported). Robust errors, reported in parentheses, are clustered at the bank-firm (pair) level

*** *p* < 0.01, ** *p* < 0.05, * *p* < 0.1, respectively

Overall, the aforementioned analyses obtained by using alternative measures to proxy trade credit leave our main findings unaffected, thus confirming the relevance of trade credit as an alternative source of external finance to spur capital investment (Carbo-Valverde et al. 2016) and further corroborating the novel evidence provided in this paper that relationship banking provided by Italian cooperative banks weakens the trade credit influence on firm investments, thus acting as a substitute for trade credit.

## Conclusions

Besides bank lending, evidence has shown that trade credit can be considered to be the next most important source of SME external financing (Wehinger 2014). Within this context, this paper provides a novel contribution to the literature by investigating the extent to which SME reliance on the trade credit channel to finance investment decisions is affected by the structure of the local banking system and relationship banking features. In particular, the novelty of our study consists in the focus on the relationship lending factors. Rather than separating sample firms based on financial (borrowing) constraints, we do so based on whether there are many cooperative banks in the province or whether bank-firm relationships are strong. We use financial data from Italian SMEs and small cooperatives banks in the country, and explore the heterogeneity in the presence of cooperative banks across Italian provinces. Specifically, we address this research questions by focusing on a sample of 6480 Italian SMEs operating with their main financing cooperative bank over the period 2008–2014 and by exploiting the geographical heterogeneity of the Italian banking market characterized by several provinces populated by an abundance of cooperative banks’ branches mostly relying on soft information in their credit relationships.

Firstly, we find a significant influence of the trade credit channel on firm investment decisions, suggesting that trade credit significantly affects the growth rate of firm investment, exerting real effects in the economy.

Secondly, we document that those SMEs located in Italian geographical provinces characterized by an abundance of cooperative banks’ branches relying on soft information-intensive relationship banking are less dependent on trade credit to finance their investment decisions. This is supportive of the view that local relationship cooperative banks still have competitive advantages over nationwide banks in small business lending, since the latter are characterized by organizational complexity and face more severe communication frictions due to the greater distance between their headquarters and local branches. This result is of particular relevance in light of the technological progress and deregulation in the banking sector that push towards eroding the comparative advantage of local relationship banks in serving small businesses.

Lastly, we provide evidence that shorter firm-bank relationships lead to a greater dependence of companies on the trade credit channel to boost investments, while the influence of firm-bank geographical proximity on this nexus is not significant due to the local nature of SMEs-cooperative banks credit relationships nurtured predominantly on a local basis.

To conclude, our results suggest that the trade credit channel plays a significant influence on firm investment decisions and that the magnitude of this influence depends on the structure of the local banking market and on the intensity of relationship banking. Since investments play a crucial role to boost SMEs’ economic recovery, the results of this paper contribute to the current academic and policy debates on safeguarding and preserving business continuity in the midst of the current Covid-19 crisis, which is likely to drive many businesses into bankruptcies. Given the profound implications of this Covid-19-induced pandemic, fostering a deep understanding of the real effects of firm financing sources is paramount to avoid bankruptcy as it can not only support financially distressed companies to benefit from policy measures aimed at preserving firms’ relationships along the supply chain, but also it can make firms more confident to invest under stressed scenarios. Moreover, this study paves the way for future research in this field. Indeed, our results call for further investigation of the substitutability between relationship banking and trade credit in funding SMEs' capital investment during the recent covid pandemic to check for the existence of different equilibria in versus out of crisis periods, for the examination of different banking institutions other than cooperative banks as well as for the analysis of larger firms to investigate whether relationship banking still weakens firm reliance on trade credit to finance capital investment for larger and more transactional borrowers.

## Footnotes

1 In the US trade credit provides as much external finance as bank loans representing approximately one third of the debt of US SMEs (Berger and Udell, 2006). In the sample used by McGuinness et al. (2018), the more than two hundred thousand companies, located in 13 European countries, are net suppliers of 62 billion euros of trade credit, equal to 14% of total assets*.*

2 For Italian firms, trade credit is a very important source of finance (e.g., Agostino and Trivieri 2014): during 2003–2009, accounts payable constituted, on average, 19.6% of total assets, while accounts receivable constituted 29.2% (source: Bank for the Accounts of Companies Harmonised, available at http://www.bachesd.banque-france.fr).

3 This allows us to take into account the fact that firms manage trade receivables and trade payables concurrently to optimise performance (Ferrando and Mulier, 2013) and manage risk (Fabbri and Klapper, 2008) since these two components of trade credit exert influence on each other (Niskanen and Niskanen, 2000). That is, firms are both trade creditors and trade debtors at the same time (Burkart and Ellingsen, 2004) and, thus, simultaneously manage receivables and payables (Hill et al., 2010).

4 Cooperative banks are a key component of the cooperative movement in the credit sector, which originated in Europe in the nineteenth century as a response to the problems that small urban and rural businesses had in obtaining credit.

5 In an environment with incomplete contracts and bargaining power, Dass et al. (2015) argue that trade credit poses itself as a quality guarantee mechanism to mitigate the uncertainty of the downstream company on the quality of acquired goods. This quality can be enhanced by extra investment efforts of the upstream company. Therefore, their empirical investigation confirms this theoretical prediction by suggesting that trade credit, in the form of accounts receivables, is positively associated with relationship-specific investments.

6 For SMEs, trade credit forms a substantial part of their balance sheet, i.e., in our sample, on average, firms engage in providing and receiving trade credit at the levels of 39.3% and 28.9% respectively, as a share of their total assets.

7 Out of these 100 Italian provinces, 43 are located in Northern Italy, 21 in Central Italy, and 36 in Southern Italy and on the islands.

8 This classification is the national version of the European nomenclature, Nace Rev. 2, published in the Official Journal of 20 December 2006 (Regulation (EC) no 1893/2006 of the European Parliament and of the Council of 20 December 2006).

9 Given that it is often not encouraged to reject outliers, especially when there is no tangible explanation about the occurrence of outliers as in the context of our paper, one remedy suggested by the related literature is to lower the impact of the outlying observations by implementing winsorization (Dixon & Tukey, 1969; Dixon & Yuen, 1974), a popular accommodation method to reduce the weights of outliers by replacing them with a specific percentile of data-dependent values (Dixon & Yuen, 1974; Orr et al., 1991). The percentile of winsorization is suggested to be adjusted according to the shape of the distribution (Dixon & Yuen, 1974; Tukey, 1962). Therefore, to minimize such influence, data are winsorized at the 5% level, i.e., 5% in each tail, following focused visual inspection of the distribution of our sample values in order to minimize the disproportionate influence of outliers on statistical analyses that might lead to distortions of statistical significance tests.

10 In unreported regressions, available upon request, we adopt an alternative cutoff point to construct *cooperative banking* according to which we set the latter equal to 1 if the density of cooperative banks’ branches with respect to the number of firms operating in a given Italian province is above the 75^th^ percentile of the distribution and 0 otherwise. Our main results remain unchanged. Moreover, our findings hold even if we cluster standard errors at the province level (as *cooperative banking* is at the province level).

11 In the study of Berger and Udell (2002), relationship banking is defined as a lending technology which is alternative to transaction banking based exclusively on hard information; in this respect, they affirm that: *“the information gathered over time has significant value beyond the firm’s financial statements, collateral, and credit score, helping the relationship lender deal with informational opacity problems better than potential transaction lenders”.* Relationship banking is based on soft information. In this respect, the figure of the loan officer is of crucial importance in collecting soft information since the loan officer not only establishes the firm-bank relationship in the first place, but also conducts due diligence during loan underwriting and performs subsequent monitoring of the borrower after the loan is disbursed (Berger and Udell, 2006).

12 Agarwal (2010) and Agarwal and Hauswald (2010) make use of the binary variable *repeated relationship* in their empirical analysis, equal to 1 in there is a prior relationship and 0 otherwise, as well as of the variable *borrower-to-branch distance.* Agarwal (2010) investigates the effect of physical firm-bank distance on the collection and use of subjective intelligence in informationally opaque credit markets. Agarwal and Hauswald (2010) examine how the allocation of authority inside the bank affects the production, transmission, and strategic use of soft information in lending decisions. Agarwal (2010) shows that greater bank-firm distance erodes the quality of local soft information, therefore reducing the reliance on soft information in credit decisions. Alessandrini et al. (2009) find that smaller operational distance does not always enhance credit availability.

13 The impact of explanatory variables on the growth rate of firm investment is computed using the “margins” command in Stata, keeping all the other variables at the average.

14 A likely scenario is the one in which weak banks may be unable to provide sufficient credit to the corporate sector to finance firm investment projects. In such a scenario, the variation in the firm investment levels explained by the corresponding bank-specific situation materializes through the corporate funding structure, which is related to the use of the trade credit channel.

15 Moreover, to mitigate regional omitted variable bias concerns due to the great heterogeneity across Italian provinces, in unreported regressions we test all our models using a more micro provincial FE setting. Specifically, in the regressions of Table 3, that would leave little variation to the variable *cooperative banking* as only its time-variation would remain, but since the interest is in the interaction variable *NTC *cooperative banking*, we don’t lose statistical power to infer its coefficient. Overall, our main findings remain qualitatively and quantitatively unaffected.
